# Understanding Events by Eye and Ear: Agent and Verb Drive Non-anticipatory Eye Movements in Dynamic Scenes

**DOI:** 10.3389/fpsyg.2019.02162

**Published:** 2019-10-10

**Authors:** Roberto G. de Almeida, Julia Di Nardo, Caitlyn Antal, Michael W. von Grünau

**Affiliations:** ^1^Department of Psychology, Concordia University, Montreal, QC, Canada; ^2^Department of Linguistics, Yale University, New Haven, CT, United States

**Keywords:** situated language processing, visual world paradigm, eye movements, verb meaning, event comprehension, sentence comprehension, language-vision interaction, modularity

## Abstract

As Macnamara (1978) once asked, how can we talk about what we see? We report on a study manipulating realistic dynamic scenes and sentences aiming to understand the interaction between linguistic and visual representations in real-world situations. Specifically, we monitored participants’ eye movements as they watched video clips of everyday scenes while listening to sentences describing these scenes. We manipulated two main variables. The first was the semantic class of the verb in the sentence and the second was the action/motion of the agent in the unfolding event. The sentences employed two verb classes–causatives (e.g., *break*) and perception/psychological (e.g., *notice*)–which impose different constraints on the nouns that serve as their grammatical complements. The scenes depicted events in which agents either moved toward a target object (always the referent of the verb-complement noun), away from it, or remained neutral performing a given activity (such as cooking). Scenes and sentences were synchronized such that the verb onset corresponded to the first video frame of the agent motion toward or away from the object. Results show effects of agent motion but weak verb-semantic restrictions: causatives draw more attention to potential referents of their grammatical complements than perception verbs only when the agent moves toward the target object. Crucially, we found no anticipatory verb-driven eye movements toward the target object, contrary to studies using non-naturalistic and static scenes. We propose a model in which linguistic and visual computations in real-world situations occur largely independent of each other during the early moments of perceptual input, but rapidly interact at a central, conceptual system using a common, propositional code. Implications for language use in real world contexts are discussed.

## Introduction

How can we talk about what we see? This question, as posed by Macnamara (1978), epitomizes a fundamental problem in human cognition: how we integrate multiple sources of information–different sensory data competing for limited attentional resources–into coherent representations of the surrounding world. The integration between sentences and dynamic real-world scenes, more specifically, depends on a system that can rapidly compute representations of very different kinds. For instance, while the linguistic input system processes phonological, morphological, and syntactic representations, the visual input is dedicated to lines, colors, textures, shapes and other more complex properties of the visual world such as objects and scene layouts. Yet, somehow these types of representations need to “talk” to each other in the process of comprehending what is seen and heard simultaneously. But how do we accomplish such a task within milliseconds of perceiving sounds and lights? How are these seemingly complex and arguably different kinds of representations put together?

**FIGURE 1:**
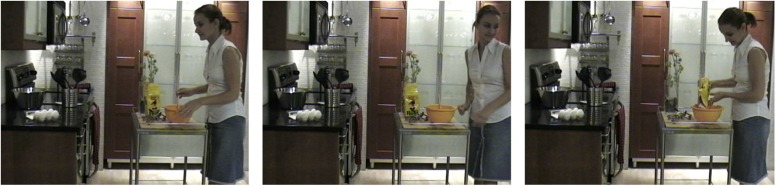
Sample frames from a dynamic scene accompanied by the sentences *Before making the dessert, the cook will crack the eggs*… (denoting a causative event) or *Before making the dessert, the cook will examine the eggs*… (denoting a perception/psychological event). The three frames represent the onset of three motion conditions of the agent of the event (the cook) with respect to the target object of the sentence (the referent of the *Theme* complement of the verb; the eggs on the kitchen counter): moving toward it, moving away from it, or remaining neutral. See text for discussion, in particular Method (Written informed consent was obtained from the depicted individual for the publication of this image).

We approach these questions by investigating, more specifically, how verbs belonging to different semantic classes, and embedded into sentences, might influence eye-movements to verb-related objects in real, dynamic scenes. In our manipulations, particular objects placed in scenes were always the referents of the complements of the main verbs in the sentences. We employed verbs from two syntactic and semantic classes, one highly constraining regarding the objects it selects in the scenes (causatives such as *crack*, *bend*) and another, non-constraining (perceptual/psychological verbs such as *look*, *inspect*). In addition, we manipulated the nature of depicted events by having agents in the scene moving in different directions–away, toward, or remaining neutral regarding those main objects (see Figure 1).

We assumed that the timing of the interaction–that is, the point at which viewers are programming an eye movement to a verb-related object during the speech stream–might indicate the level at which vision and language exchange information. Specifically, if the two systems are encapsulated (or modular, in the sense of Fodor, 1983, 2000; see also Chomsky, 2018), then they should make the products of their linguistic and visual computations available to a common, higher conceptual system. This would result in delayed saccades to objects as a function of verb type and agent-motion type, thus reflecting a late, conceptual interaction. Conversely, if the two systems freely exchange information during their respective early inputs–thus, if they are interactive (McClelland et al., 1986, 2014)–then saccades to verb-related objects should be observed at an early point during verb processing, even in anticipation of the name of the objects attracting saccades, as some studies have suggested (e.g., Altmann and Kamide, 1999; Staub et al., 2012).

These different empirical predictions rely on the widely held assumption that eye movements to particular visual targets are largely under the control of linguistic variables. This assumption comes mostly from studies employing the so-called visual world paradigm (VWP), which involves the concomitant presentation of visual and linguistic stimuli while viewers/listeners have their eyes monitored by an eye tracker (see, e.g., Tanenhaus and Spivey-Knowlton, 1996). A key issue underlying this paradigm is the potential influence of visual context on linguistic processes, as revealed by patterns of eye fixations and scan paths. The use of this paradigm to investigate the architecture of the language-visual interaction raises important questions on the nature of the representations employed in the task of processing sentences and scenes simultaneously. It is of general agreement in cognitive science that for the two systems to influence each other they need to transform their input representations into a common format (Fodor, 1975; Macnamara, 1978; Jackendoff, 1987, 2012). A key question underlying our investigation is whether this common representation format affects early input processing or whether it affects only later stages of processing when both systems have delivered their respective input analyses to a common, higher conceptual system.

In the next section, we first review selected studies that have investigated the interaction between language and vision using the VWP, focusing on the interaction between verb information and objects/referents in scenes or displays. We address methodological issues with these selected studies, which have motivated our empirical investigation employing more naturalistic stimuli. Then, we discuss the proposals for how the two systems might interact. Following this preliminary discussion, we report our eye-tracking experiment involving different verb classes and dynamic scenes. We are particularly interested in how this study can inform us about the architecture that serves the language-vision interaction. To that end, we further develop our proposal for the nature of the representations that afford the interaction between linguistic and visual information in the General Discussion section.

## The Visual World Paradigm and the Architecture of the Language-Vision Interaction

### Visual World Studies

Although the workings of language and vision have been the topic of much research and have figured prominently in integrated models of cognitive processes (e.g., Jackendoff, 1987; Potter, 1999, 2018; Baddeley, 2012) the investigation of how the two systems might influence each other has been somewhat neglected up until recently. While Baddeley’s (2012) working memory model, for instance, postulated different buffers for visuo-spatial and phonological processes, it was Jackendoff (1987) who first proposed a model of how visual and linguistic systems might combine their respective representations. But it was the development of the VWP (Cooper, 1974; Tanenhaus et al., 1995) what motivated numerous studies on the interplay between language and vision (see Huettig et al., 2011, for a review). These studies have raised new questions on how visual and linguistic computations interact in the process of understanding what we see and hear simultaneously, with most studies focusing on how information presented in static displays might influence the course of linguistic computations, such as the resolution of temporary lexical, syntactic, or semantic ambiguities. An important advantage of this technique is its ecological validity, for, depending on the variables manipulated, it may closely mimic how linguistic utterances might unfold in visual contexts.

The potential influence of visual contexts on linguistic operations was investigated in Tanenhaus et al.’s (1995) pioneering studies (see also Spivey et al., 2002) involving participants manipulating real objects while following spoken commands that contained temporary syntactic ambiguities such as *Put the apple on the towel in the box*. Relevant to our study is the timing of the participants’ saccades in relation to the target word (*apple*). In the critical visual conditions, when there was a contrast between one-referent (an apple on a towel) and two-referent contexts (an apple on a napkin and an apple on a towel), the pattern of saccades to and out of the referent region (the apple) were relatively slow. In the one-referent context, subjects looked to the apple about 500 ms after hearing the word *apple*. And in the two-referent context, subjects looked to the correct apple 50% of the time, about 1100 ms after hearing the word *apple*. This study was taken to speak against the view that syntactic structuring–including verb-argument structure–underlying interpretation is an autonomous process, relying primarily on syntactic parsing principles (see, e.g., Fodor, 1983; Frazier, 1988).

This study, however, should be interpreted with caution. First, it had few subjects (six; see also Spivey et al., 2002). Second, subjects were given the opportunity to watch the placement of objects on the table, thus previewing the nature of the incoming displays for “a few seconds” (p. 460), which could have given participants the opportunity to conceive of several possible ways for the task to unfold. More importantly, the timing of saccades reported in the study seems incompatible with the view that subjects are integrating visual context early on during sentence processing. While its possible that delays in the two-object condition may reflect true effects of context on early *parsing* decisions, it is also possible that they reflect late, problem-solving strategies.

Numerous other studies involving this paradigm have manipulated different linguistic variables (words, sentences) and visual materials such as color line drawings (clip art) of scenes (e.g., Altmann and Kamide, 1999; Kamide et al., 2003; Knoeferle et al., 2005) photographs of scenes (Andersson et al., 2011; Staub et al., 2012; Coco et al., 2016), and sets of photographs of people and objects (e.g., Boland, 2005). In the remainder of this section, we restrict our discussion to specific issues on how *verbs* might direct attention to referents of their arguments, but we do so based on a few selected findings that are more directly related to the present study. The relationship between a verb and the referents of its arguments in a scene is of particular concern not only because of its connection with the experiment we report below, but also because it speaks more directly to our main theoretical concern: how representations from vision and language interact such that we can understand and produce utterances referring to what we see.

Altmann and Kamide (1999) employed the VWP to investigate sentence interpretation as a function of different verb selectional restrictions, that is, the types of real-world objects that verbs semantically select (e.g., “something edible” for the verb *eat*). Using ersatz scenes (Henderson and Ferreira, 2004), they found that saccades to the drawing of an object such as a cake were faster when the accompanying sentence was *The boy will eat the cake* than when it was *The boy will move the cake*. Saccades to the picture of the cake occurred on average at about 200 ms (*eat*) and 400 ms (*move*) after the offset of the verb. Eye movements for the constraining condition (*eat-*cake) were said to be *anticipatory*, i.e., they occurred before the noun onset. In their Experiment 1, they found that anticipatory fixations to the object (e.g., cake) occurred in 54% of the trials in the related condition (*eat*), compared to 38% of the trials for the unrelated (*move*) condition. These effects, measured by saccade-to-object onset times and percentage of trials, were obtained when participants were told that they had to judge whether or not objects in the scene were mentioned in the sentence. In the absence of an explicit judgment about sentence/scene match (Experiment 2), the effects were reduced but consistent with the first experiment (32 and 18% in the related and unrelated conditions, respectively). They suggested that what is accessed at the verb “is not structure *per se*, but *interpreted* structure” (p. 259). That is, information about a scene is assimilated by–or interacts with–the ongoing sentence interpretation process such that a *semantically related* argument of the verb has advantage over an unrelated one.

This study was further supported by Staub et al.’s (2012). Employing photographs of scenes, they also found anticipatory eye movements to target objects for semantically constraining verbs (*eat*) but not for control verbs (*move*). There were, however, several differences between the two studies, including method and statistical analyses. For instance, contrary to Altmann and Kamide, Staub et al.’s (2012) sentences did not contain a 200 ms pause between the verb and the determiner. In Altmann and Kamide’s study this could have yielded earlier saccades in the related (*eat-*cake) condition. But it should be noted that Staub et al.’s (2012) results, on the other hand, were obtained with sentences that differed in length: while the relevant constraining segment (*eat the*) took 617 ms, the non-constraining one (*move the*) took 688 ms on average. Assuming that “express saccades” (see Fischer and Ramsperger, 1984) to targets can be obtained at about 100 ms, it is possible that the 71 ms length difference between the two conditions could have given the constraining condition a head start, producing faster saccades to target objects.^1^ Indeed, the mean difference in saccade latency between the two conditions–105 ms–is close to their difference in length. Also, percentage of fixations on the target object differed significantly in the two verb conditions, but they were as small as 5% by verb offset and 7.5% by noun offset.

Coco et al. (2016) also offered a quasi-replication of Altmann and Kamide (1999) employing pictures of scenes with sentences containing constraining and unconstraining verbs, such as *The man ate/removed the sandwich*. Additionally, the referent of the internal argument of the verb (*sandwich*) was present or absent in the picture.^2^ They did not report latency of saccades, and used part of the scene (e.g., a table) as the region of fixations in lieu of the object. They found a greater proportion of fixations on the “contextual object” (table) with the constraining verb than with the unconstraining one, in a window of time that spans from 100 to 700 ms post verb onset. The difference between the two verb conditions was also significant in the object absent comparison. These results suggest that subjects took the verb information and anticipated a plausible referential location for the upcoming (or unfolding) target object name. It is, however, difficult to contrast this study directly with the others such as Altmann and Kamide (1999) and Staub et al. (2012), for two main reasons: (1) the lack of latency data, and (2) the nature of the data reported, which does not allow us to determine the magnitude of their effects–neither at particular time points after verb onset, nor overall, up to noun onset.^3^

While Coco et al. (2016) controlled for possible word association confounds, it is not clear whether the effects reported in both Altmann and Kamide (1999) and Staub et al. (2012) are due to verb structure (viz., argument or thematic structure) or simply semantic relatedness between verbs and more plausible objects in the context, such as *eat*-cake. This difference is important because if what is at stake is semantic relatedness–a form of priming–it could be argued that the effect does not reflect *influence* of visual context on early linguistic computations, but a late, conceptual effect. Kamide et al. (2003), however, suggest that indeed effects of verb-argument structure are involved in the process of incremental interpretation. In their Experiment 1, employing double object constructions such as *The woman will spread the butter on the bread/The woman will slide the butter to the man*, with scenes depicting the four referents (man, woman, bread, butter) they found that there was a greater proportion of trials in which participants looked at the “appropriate” *Goal* (*bread* in *spread*; *man* in *slide*) than in the “inappropriate” *Goal* (*bread* in *slide*; *man* in *spread*). These effects were not obtained at the verb region (a window of 350 ms post-verb onset) but during the processing of the *Theme*, the noun *butter*. Their verb effects in the *Theme* region, which is a window of 882 ms during the processing of *the butter*, were small (and non-significant, in one of the analyses). This was taken as evidence that listeners anticipate an appropriate *Goal* for a ditransitive verb such as *slide*, but not for a verb such as *spread*. They supported a view of language processing in context that takes into account “all the syntactic, semantic, and real-world constraints that can be applied” (p. 153) at a given segment, attempting to predict the nature of other potential arguments.

Knoeferle et al. (2005) have also investigated the interaction between depicted events and verb-argument processing. In one of their studies (Knoeferle et al., 2005), they presented three characters (e.g., a princess, a pirate, and a fencer) as an ersatz scene while participants heard sentences referring to their roles (e.g., *The princess is apparently washing the pirate* [Subject-Verb-Object]/*The princess is apparently painted by the fencer* [OVS]). In their Experiment 1, they found greater inspection (proportion of looks) of the object (e.g., the pirate) upon hearing the verb. The effect was obtained at about 2000 ms from the onset of the sentence and before the grammatical object was uttered. Notice, however, that the scenes had only three characters: the one in the center was the princess, which could be understood as subject or object, thus there was a 50% chance that one of the two remaining characters would be looked at once the princess was inspected. Proportions of looks to the appropriate character, however, were below chance during the verb, with about 40% of the looks into the princess up until the adverb onset. This study was taken to support the idea that “non-linguistic information–such as contrast, actions, or events–that establishes relevant relations between entities, can affect how linguistic input is interpreted” (p. 122).

In summary, the studies briefly reviewed in this section claim to provide strong support for the view that information about the visual context aids linguistic processes of syntactic structuring (argument/thematic structure) and semantic interpretation. The language comprehension system is said to be incremental, at each moment considering *all* available sources of information–and in particular, verb-related information such as the conceptual nature of arguments. And it is because language use normally occurs in visual contexts that those studies supposedly carry a high degree of ecological validity, bearing on the nature of cognitive processing architecture: the weight of the evidence seems to favor a highly interactive and probabilistic rather than a modular, rule-based view of language comprehension in visual contexts.^4^ However, as we pointed out, there are numerous methodological issues with these visual world studies, casting doubt on their generalizability.

### Problems With the Visual World

One of the main problems with the visual world studies reviewed above is the timing and nature of saccades reported. For the most part, saccades to relevant objects are relatively late, when other linguistic constituents are being processed (as in Tanenhaus et al., 1995; Spivey et al., 2002); or they occur after an artificially introduced break in the sentence (Altmann and Kamide, 1999); or they are triggered by linguistic segments of unequal length (Staub et al., 2012). Moreover, the proportion of fixations that are usually reported as evidence of anticipatory eye movements to targets is relatively small, often below chance. Consider, for instance, Kamide et al.’s (2003) Experiment 2. They report anticipatory effects in a region spanning 637 ms after verb onset with looks to relevant targets occurring in only 10% and 7% of the trials in the experimental and control conditions, respectively (a statistically significant effect). What is perhaps most surprising in this study is that looks to other regions of the scene occur in 55% of the trials with agents receiving 35% of all fixations, an amount greater than any of the target objects they were contrasting. Thus, although verb-scene semantic effects were found, most eye movements do not appear to be locked into the initial process of interpreting arguments and their referents in the visual world.

A second methodological problem with these studies is the nature of the visual context they use. Given that they do not involve realistic dynamic scenes, they can only generalize to language use in static contexts, limiting the strength or their challenge against modular systems. One possible interpretation of their results is that the lack of agents and motion in depicted events frees attentional and gaze mechanisms to be controlled by linguistic processes of interpretation, thus yielding effects of anticipation. Notice that this does not rule out that early interference of visual context on linguistic processes might be exerted–but they might occur under relatively artificial conditions, thus weakening the claims that vision and language are interactive tout court. Moreover, given the timing of saccades and the small proportion of early *fixations* (which occur after targets are encoded and saccade programming occurs) the effects obtained by visual world studies are compatible with a view that takes language and vision to be initially encapsulated, but interacting at higher processing levels (see below). Knoeferle et al.’s (2005) visual contexts, for instance, require participants to *infer* that a princess would be holding a bucket with the purpose of washing a pirate; or that a princess would be holding an artist’s brush and palette to be painted by a fencer. Not only do the scenes look unnatural, the level of detail required to make the appropriate inferences about the characters’ roles may account for the high percentage of fixations (about 40%) to the princess, the central character, during verb processing and up to adverb onset.

A third reason for questioning the claims stemming from the visual world literature is the nature of the representations and the interaction mechanisms proposed. The main assumption is that visual and linguistic processes interact early on, possibly at perceptual levels of analyses. In fact, most studies supporting interactionism have lined up with a cognitive architecture that postulates no clear distinction between levels of analysis or processing components (e.g., Altmann and Kamide, 2007; Mayberry et al., 2009; Smith et al., 2017). Smith et al. (2017), for instance, take the constraints exerted between semantic and visual processes over linguistic/phonological processes to be a function of co-activation of nodes in a connectionist network. But, this only begs the question about what nodes stand for and how links are obtained in the “integrative layer” (viz., how a “phonological” node talks to a “visual” node). Besides these nodes being arbitrarily determined in terms of both the *content* they stand for and the nature of the links that obtain between them at different layers, a perennial problem with connectionist models of the semantic system is that they cannot account for the *compositionality* and *productivity* characteristic of linguistic and conceptual systems (see Fodor and Pylyshyn, 1988). Mayberry et al.’s (2009) CIAnet is also a connectionist model based on Knoeferle and Crocker’s (2006) coordinated interplay account (CIA, for short). It incorporates attentional mechanisms that are responsible for activating “the event in the scene most relevant to the utterance” and it does so by “learning to bind the events’ constituents” (Mayberry et al., 2009, p. 462). While CIA (and CIAnet) is explicit about some of the representations computed (the likes of *Agent*, *Action*, *Patient*), it does not make explicit how vision extracts that information from the scene, other than assuming that visual inspection produces event interpretations and predictions. Contrary to these models, the architecture we propose below is committed to a common representation format for the interaction between language and vision, relying on the independent and parallel computation of both systems at the earliest stages of processing during dynamic scene and sentence comprehension.

### The Nature of Representations and the Architecture of the Language-Vision Interaction

In the present study, we manipulated realistic dynamic scenes and sentences aiming to understand the interaction between linguistic and visual representations in real-world situations. Consider, for instance, witnessing one of the events depicted in Figure 1 while listening to a sentence such as *The cook will crack the eggs that are in the bowl*. Upon hearing the causative verb *to crack*, there are only a few objects in the scene that might be relevant for understanding the unfolding event–the *eggs* among them. Consider now the same sentence with a perceptual/psychological verb such as *to examine*. While *crack* restricts the potential referents in the scene, *examine* allows for a wider range of objects, as possibly anything in the scene can be examined. Moreover, while the causative class denotes a change of state in an object in the scene (the referent of the *Theme*), the psychological class might denote a change of state in the agent (the *Experiencer* of the event). Therefore, these verb types represent a clear contrast with regards to the relation between an agent (typically, the sentence’s grammatical subject) and a real-world object. As we have seen above, several visual world studies using ersatz or static pictures of scenes have suggested that verbs lead to *anticipatory* eye movements to the referents (i.e., the objects) of their noun complements when a given relation between a verb and potential referent is established. Although there are numerous methodological differences between these studies, as well as different interpretations for their results, the effect has not been generalized to realistic, dynamic scenes, which arguably better represent “how we talk about what we see.”

One of the problems with static scenes is that they are predictable and thus might not tax the attentional system, allowing the eyes to move freely, promoting eye movement behavior that is more likely to be in consonant with the linguistic utterance (but see Andersson et al., 2011). Dynamic scenes, however, while often predictable due to physical constraints (e.g., inertia and gravity), also have a high degree of unpredictability in particular when human agents are involved. As shown in Figure 1, there are numerous possibilities with regards to what the agent might do while remaining true to either the …*crack*… or …*examine*… versions of the sentence. We can assume that at least three actions might take place: the agent may move toward the object, she may move in the opposite direction, or she may remain neutral (i.e., continue mixing the dough). Would different agent behaviors affect what the viewer attends to? And, more specifically, would attention be drawn to target objects (*eggs*) independent of and in anticipation to agent action in the case of the more restrictive causative verb? If, as studies have suggested, attention is driven to objects *automatically* and in *anticipation* of the noun being heard, we should expect that agent action should not affect saccades to their selected objects; along the same line, we should expect a verb effect to be obtained, with the more constraining verb (*crack*) always leading to faster saccades to the referent of its complement noun (*eggs*).

While these questions about the role of verb restrictions and agent action in dynamic scenes are important for understanding how linguistic and visual processes might influence each other, equally important are the processes that allow for the combination of representations from both input streams. In Jackendoff’s (1987, 2012) model, linguistic and visual inputs run parallel, independent processes analyzing their respective stimuli. Their outputs reach a central, conceptual structure system, after they are translated via interface modules and coded into a propositional form, compatible with both, the representation of the spatial structure on the visual side and the representation of the structural properties of the sentence, on the linguistic side.^5^ This conceptual system, more importantly, operates on a symbolic, amodal code that is common to the *products* of both input systems, language and vision.

**FIGURE 2:**
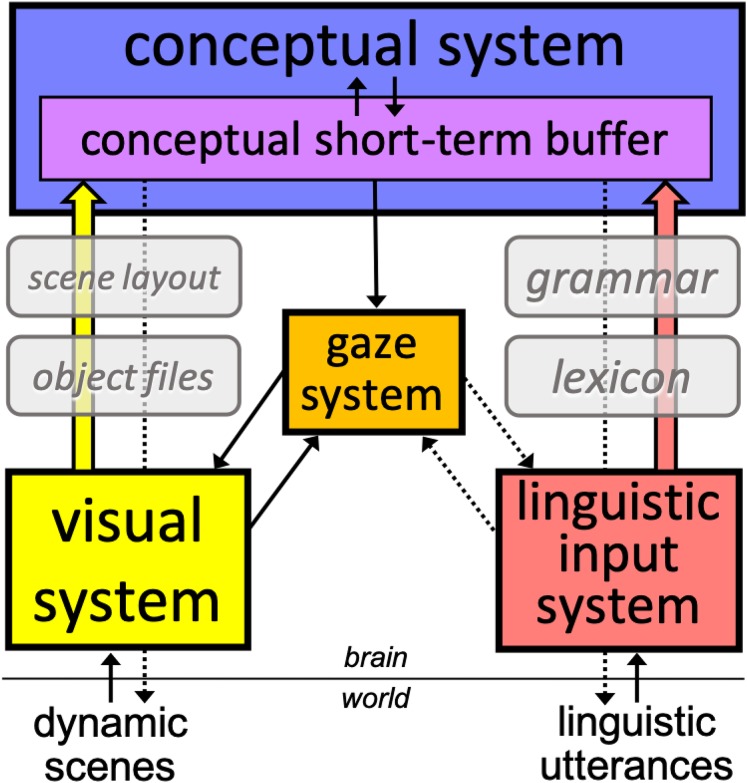
A schematic representation of the proposed modular processing architecture bearing on the interaction between language and vision. The solid arrows represent the mandatory flow of information from input systems to the conceptual system and from this to the gaze system. The dotted arrows represent hypotheses about top-down attentional mechanisms involved in (a) locking into referents in the world (e.g., tracking objects, updating representations), (b) attending to the spoken source, (c) tracking low-level properties of linguistic analyses (e.g., computing phrase structure), and (d) reading mechanisms (e.g., word fixation and next-word saccade programming). The conceptual short-term buffer integrates predicate-argument relations computed from both systems, relying on the input systems’ knowledge of objects and scene layouts, on the visual stream, and the lexicon and the grammar, on the linguistic stream. See section “General Discussion” and Supplementary Figure S2 for a more detailed presentation of the workings of the model.

Our hypotheses stem from a parallel modular architecture that is closely aligned with Jackendoff’s (2012) model. As Figure 2 shows, we assume two main autonomous input systems that feed a central conceptual system, which dynamically updates accessed representations in conceptual short-term buffer (CSTB). CSTB actively combines concepts into propositional structures computed from both linguistic utterances and dynamic scenes. While predicates and arguments constitute the basic building blocks of linguistic-semantic representations, they have also been proposed to constitute the fundamental representations of visual processes (Pylyshyn, 2007). These predicates are primarily descriptive of object and scene properties (the “arguments”) such as spatial relations and scene dynamics (e.g., trajectory of objects and agents). The gaze system programs and executes saccades based primarily on input from the visual system (exogenous), and conceptual-structure representations (endogenous), including schemas such as those deployed in the control of voluntary activities (see, e.g., Land, 2009). The gaze system also responds to linguistic input, in particular lexical properties, which determine patterns of saccades and fixations in reading (Reichle et al., 2003). Crucial to the interaction between the two systems is the allocation of attention to scenes and to the linguistic input. Visual attention locks into multiple objects in the visual field, which are the primary sites for potential fixations during scene and sentence processing.

While details of this model’s operations are beyond the scope of the present introduction (see section “General Discussion” and Supplementary Figure S2, for further description), it is important to highlight its predictions for the simultaneous processing of visual and linguistic representations.

First, we predicted that the more restrictive type of verb, causative, would lead to faster saccades to the object, compared to the less restrictive perception verb. We assumed that if there are anticipatory saccades to the target object, the nature of the object that matches the semantic restrictions of the verb has to be already encoded or activated. This would imply that viewers/listeners would have established early on a connection between verb meaning and object meaning. This connection would also reflect in anticipatory saccades to target objects across scene types, but with greater effects in the toward condition, compared to neutral and away conditions. By contrast, a lack of anticipatory eye movements could be seen as the eye-gaze system’s reliance primarily on visual processes of scene comprehension, over and above the potential effects of verb-semantic restrictions. In other words, one should expect saccades to the target object to reflect a late assessment of *both* visual context and verb-semantic restrictions, such that only upon hearing the referent of the internal argument *and* evaluating the scene semantics that saccades to target objects would occur. This prediction would rule out verb-driven anticipatory effects, but not motion effects, with toward condition yielding faster but non-anticipatory effects to the target, independent of verb type. We should note that, as pointed out by a reviewer, it is possible to conceive of a late interaction to be driven by factors such as the cognitive load brought about by the complexity of the scene (see, e.g., Andersson et al., 2011) in lieu of architectural restrictions. We contend, however, that related studies using similar naturalistic scenes as ours (e.g., Staub et al., 2012), though without motion, did report anticipatory eye movements. If scene motion is a factor we should expect an interaction between verb type and agent motion, signaling verb-driven effects on eye movements to related objects with a causative-verb advantage. Moreover, our toward condition should *promote* anticipatory eye movements to the referents of causative verb complements. This is so because, contrary to other studies with static scenes, participants have two sources of information to drive their potentially anticipatory looks: verb restrictions and agent motion. Ultimately, the present study addresses a methodologically important point which is the reliability of the VWP to make claims about the architecture of the language-vision interaction insofar as realistic motion events are concerned.

## Methods

### Participants

Thirty-eight Concordia University students (32 females) participated in the eye-tracking experiment, none of them participated in the norming studies developed for the preparation of materials. Participants were all native speakers of English and had normal or corrected-to-normal (with contact lenses) vision. Their age ranged between 19 and 38 years of age (*M* = 22; *SD* = 3). They all gave written informed consent and participated for course credit as part of the Concordia Psychology Participant Pool. The experiment was approved by the Concordia University Human Research Ethics Committee.

### Materials and Design

#### Verbs

We initially selected 18 lexical causatives (e.g., *crack*, *bounce*), and 18 verbs that were either perception (e.g., *see*, *hear*) or psychological verbs (e.g., *examine*, *notice*), following Levin’s (1993) classifications. Only 17 of each kind were used in the eye-tracking experiment due to a movie recording error which led us to eliminate one trial before we conducted the experiment but after all materials had been prepared. Norms for verb complements were obtained from a study with 58 Concordia University participants, all native speakers of English, who did not take part in the main experiment. These participants were required to fill-in simple sentence frames such as *The man bounced the _________* and *The ________bounced*. The complements used in the sentences and movies were chosen based on three criteria. (1) They were the nouns most frequently given in the frames. (2) They were not the strongest associates of the verb. This association was determined by 10 other participants who were required to provide the first word that came to mind for each verb. Thus, if for a frame such as *open*-____ the most frequent associate provided was *door*, we eliminated *door* as a complement for *open*. And, (3) the referent objects constituted mid-size objects, which could be filmed in mid-ground. Therefore, we eliminated cases such as *wall* for *crack* (the chosen was *eggs*). The perception and psychological verbs were also determined based on Levin’s (1993) classification of verbs that take an *Experiencer* as external, subject argument, as opposed to an *Agent*. This criterion applied to at least one sense of those psychological verbs that can have multiple senses. These verbs were matched with the causatives based on frequency (Kucera and Francis, 1967; Coltheart, 1981) but also on the plausibility of the events to be filmed (to allow for pairs such as …*crack/examine the eggs*), as judged by the experimenters and two research assistants.

#### Sentences

Seventeen sentence pairs were created, with each member of a pair differing only with respect to the main verb, which belonged either to the causative or the perception/psychological class (e.g., *Before preparing the dessert, the cook will crack/examine the eggs that are in the bowl*). All sentences had an initial patch clause, which was always of an adverbial type (e.g., *Before preparing the dessert*…, *After playing with the toys*…), followed by a main clause. All main clauses were of the form NP1 (Noun Phrase1)-*will*-Verb-NP2-RC (Relative Clause). The NP1 always referred to the agent in generic form (*the cook*, *the boy*, *the man*, etc.); the NP2 referred to the target object in the scene (*eggs*, *ball*, etc.); and the RC always made reference to the target object (e.g., …*that are in the bowl*, …*that is on the bench*). It should be noted that although our sentences contain a RC with a prepositional phrase that could signal a potential disambiguation between two competing referents, this comes after the crucial complement noun, thus after the point of interest regarding verb-driven saccades to target objects. Moreover, as can be seen in our materials, in most of our scenes there is only one referent for the verb complement noun.^6^ Sentences were recorded by a female research assistant speaking at a normal pace.

#### Scenes

Each movie consisted of single shots of about 10 s of indoor or outdoor naturalistic scenes. There was no camera movement or zoom, and the only source of motion within the movies was that of the agent performing a given action. For films produced at a large furniture store, the store displays were arranged to resemble common household areas (e.g., kitchen counters were filled with utensils, bookshelves were filled with books and objects). For all the films produced at houses, parks, and streets, the only scene alterations were the placement of target objects (e.g., a kite on a park bench). Agents and target objects were on the same plane (always mid-ground) in opposition to each other (e.g., if the agent was on the left, the target object was on or near the right edge of the visible image). Each of the 17 unique scenes (e.g., someone cooking in a kitchen) was filmed with three different endings: after an initial similar segment of about 7 s, agents moved (or reached) either toward a particular target object, away from it, or remained neutral, that is, continued doing what they were doing in the initial segment (see Figure 1). There was thus a total of 51 unique movies (17 scenes × 3 endings). Each movie was then synchronized with two sentences (causative and perception/psychological), yielding 102 film/sentence combinations. The 102 stimuli were distributed in six lists of materials, each one containing 17 trials (film/sentence combinations), with two or three of each verb-type/motion-type combination. We did not use filler items noting that Staub et al. (2012) have obtained no statistically significant differences between lists of materials when they were ran with different filler types (e.g., standard, unpredictable) and no fillers. Film resolution was set at 720 × 480 pixels in NTSC format (29.97 frames per second). The digital movies were produced and edited by a Concordia film student using Final Cut Pro (Apple, Inc.). Scene norms included object saliency and scene semantics (predictability of events). These are described in Supplementary Material. Please also see Supplementary Material for sample video (written informed consent was obtained from the depicted individual shown in the video for its publication).

#### Sentence-Film Synchronization

Sentences and films were synchronized such that verb onsets in the sentences corresponded to agent action onsets in the movies. The agent action onset was determined by inspecting, frame-by-frame, the point in which the agent started moving toward or away from the target object. For each movie, there were up to seven frames in which the agent motion direction could be said to have started (e.g., beginning of rotation of the torso or limbs toward or away from target object). The investigators selected one of these frames as the onset of the action. Since each frame corresponds to 33.37 ms, the onset of the agent motion–that is, the moment the agent turns unambiguously toward or away from target object–was determined within a window of 234 ms (7 frames), with the corresponding time in the neutral motion condition. We call this the “disambiguating point” (see also Supplementary Figure S1). Acoustic onsets of the verbs in the sentences were determined by amplifying the acoustic waveforms in the digital sound files and identifying the lowest frequency marking the boundary between words, or by splitting transitional phonemes when the lowest frequency was not obtained.

### Apparatus and Procedure

**FIGURE 3:**
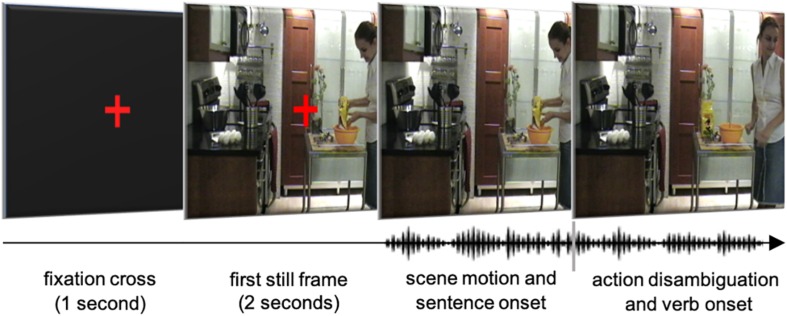
Schematic depiction of the procedure. At the beginning of each trial, participants were presented with a red cross over a black background, centered in the middle of the screen. After 1 s, the black background was replaced by the first still frame of the movie for 2 s, with the fixation cross still in place. After that point in time, the cross disappeared and the movie was set in motion. The accompanying sentences began within a few seconds after the movies began, but the actual onset time of each sentence varied, depending on the synchronization between the acoustic onset of the main verb and the frame corresponding to the beginning of the action performed by the agent in the movie–which was different for each movie triplet (Written informed consent was obtained from the depicted individual for the publication of this image).

Visual and auditory stimuli were presented via PsyScope software (Cohen et al., 1993) on an Apple computer placed 41 cm away from participants. Participants wore clip-on headphones. Their eye movements were recorded using the EyeLink-I head-mounted eye-tracker at a sampling rate of 250 Hz from the left eye only (viewing was binocular). Head movements were minimized with the use of a chinrest. Figure 3 shows schematically how stimuli were presented in each trial, from the fixation cross to the verb and agent activity onset. Participants were instructed to watch the movies and to listen to the accompanying sentences while paying close attention to both as they would later be tested with a recall task. They were asked to press the space bar on the computer keyboard to begin each trial. Each trial lasted approximately 12 s and the full experimental session lasted about 30 min, including eye-tracker calibration time.

## Results

### Data Integrity and Analysis Methods

The post-experiment recall test consisted of a booklet with 12 sentences and 12 still frames of the movies, half of which had been presented in the experiment. Participants scored between 75 and 100% (*M* = 87.6%, *SD* = 7.3%) in the cued recall task, indicating that they attended to both movies and sentences; therefore, data from all participants were kept in the analyses. Of the total trials, 4.5% were missed due to corrupted data. These trials were distributed evenly across the various experimental conditions. In 91 trials (14.3%) participants did not look at the target object. A 2 (verb type) × 3 (motion type) repeated-measures ANOVA conducted on these trials showed no effect of verb type (*F*(1,74) = 1.81, *p* = 0.19) or of motion type (*F*(2,74) < 1, *p* = 0.88), nor was there a significant interaction effect between the two main variables (*F*(2,74) < 1, *p* = 0.52). This indicates that the apparent motion of the agents in the scenes and the verb class did not affect whether or not participants fixated on the target object after verb-onset; in other words, the number of trials with no post-verb fixations was evenly distributed across the six conditions. The third source of missing data derived from trials in which participants happened to be fixating the target object at verb onset. This occurred in 41 (6.4%) of the trials. These trials had to be excluded from any analyses examining the effect of verb type on subsequent eye movement behavior because it was not possible to determine whether or not participants continued to fixate the object as a function of the verb that they heard.^7^ Again, a 2 × 3 repeated-measures ANOVA was conducted to examine the effect of verb type and motion type on the proportion of these trials (by subjects). The results indicated that the verb type failed to reach significance, *F*(1,74) = 3.84, *p* = 0.06, although there was a tendency for a larger proportion of trials to be of the causative type across all motion conditions. But there was no main effect of motion type, *F*(2,74) < 1, *p* = 0.64, nor a significant interaction effect, *F*(2,74) < 1, *p* = 0.98, as expected. These results suggest that the agent’s apparent direction of motion at verb onset did not affect whether participants were fixating the target object at the time the verb was spoken.

Analyses of the remaining data took into account three basic measurements of eye movement behavior. (a) Saccade onset time (SOT) which was the time taken by the viewer to launch an eye-movement (saccade) to the target object (always the object referent of the grammatical complement of the main verb of the sentence) after the disambiguating point. This measurement took into account all saccades and fixations made between verb onset and the final direct saccade to the target object but without counting the target fixation time; for the causative sentences, this corresponded to a window of 666 ms and, for the perception sentences, 768 ms. We took this analysis to represent a more on-line measurement of the reflexive behavior triggered by attentional grabbers in the visual scene (directed or not by linguistic cues), in comparison with often reported proportion of trials in which a fixation to a target occurred. (b) Number of saccades produced after the disambiguating point until the viewer fixated on the target object; this was the total number of saccades from verb onset including and up to the first saccade to the target object; and (c) cumulative saccades to the target object during the period following the verb-onset up to the acoustic offset of the spoken noun (the direct object of the main verb).

We relied on two data-analytic methods. First, we report repeated-measures ANOVAs taking into account participants (*F1*) or items (*F2*) as random variables, together with planned pairwise comparisons between verb types across different levels of the motion condition, also considering either participants (*t1*) or items (*t2*) as random variables. For these analyses, data from seven participants who had missing values (means) in one or more conditions were removed from all analyses. Following the removal of the seven participants, we then screened the data for potential outliers. All SOTs below 200 ms were subsequently removed, which resulted in the removal of 23 data points (4% of the total dataset). Furthermore, the Shapiro–Wilk’s test revealed that the assumption of normality had been violated for four conditions (i.e., causative-away, causative-toward, perception-away, perception-neutral) in the SOT data only. Thus, for all analyses we conducted the ANOVAs on the logged transformed data. Second, we report linear mixed effects models (LME) for all three eye-movement datasets, also with subjects and items as random effects. Although the latter analysis method has become standard in psycholinguistic experiments, we deemed the first analysis type particularly important for it would allow us to contrast our effects with those of previous studies employing the VWP with scenes and different verb types (in particular, Altmann and Kamide, 1999, and Staub et al., 2012).

For the LME analyses (Baayen et al., 2008) we employed the lme4 package (Bates et al., 2013) for the R statistical programming environment (R Development Core Team, 2012). Given that LME accounts for participant idiosyncrasies, we entered all of the participants’ raw data into the analyses. As such, the seven participants that were originally excluded in the ANOVA’s analyses, due to missing values in one or more conditions in the SOT data, were included, resulting in a total of 38 participants.

For all LME analyses, our models were fitted using a backward step-wise elimination procedure, whereby the predictor variables that did not significantly improve the model as indicated by likelihood ratio testing were subsequently removed. Furthermore, all models included only random intercepts for participants and items, as justified by the likelihood tests, given that the simple model could not be rejected in favor of a more complex model. The most complex model, which included random slopes for the fixed effects and their interaction, did not converge for any of our analyses, and thus could not be evaluated. We then derived *p*-values for each predictor variable by comparing the fitted model to a minimally contrasting null model that excluded the relevant term. Planned comparisons were conducted using the emmeans package (Lenth et al., 2018).

### Saccade Onset Time

**FIGURE 4:**
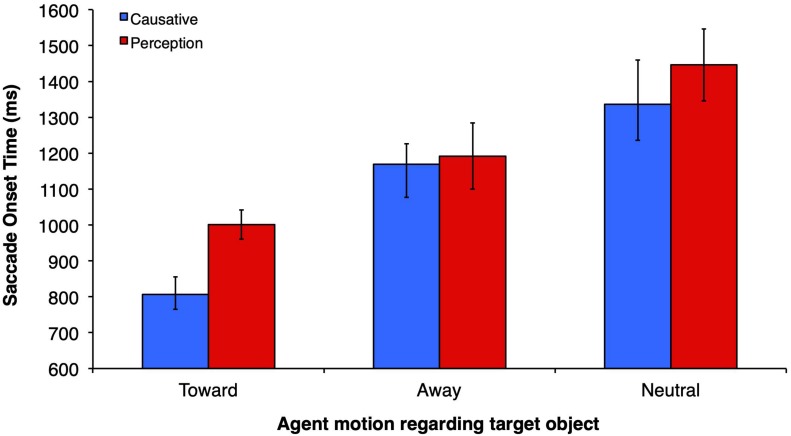
Saccade onset time (SOT) representing the mean time taken by viewers to launch a saccade to the target object after the onset of the verb.

Figure 4 depicts the mean SOT for each verb and motion condition. There was a main effect of motion type (*F1*(2,60) = 11.27, *p* < 0.0001, η_p_^2^ = 27.3, observed power = 0.99; *F2*(2,32) = 9.75, *p* < 0.0001, η_p_^2^ = 37.9, observed power = 0.972), and a marginally significant effect of verb type in the participants analysis (*F1*(1,30) = 4.16, *p* = 0.05, η_p_^2^ = 12.2, observed power = 0.506), and in the items analysis (*F2*(1, 16) = 4.14, *p* = 0.059, η_p_^2^ = 20.5, observed power = 0.481). There was no significant interaction between verb type and motion type (*F1*(2,60) = 0.31, *p* = 0.73, η_p_^2^ = 0.10, observed power = 0.10, *F2*(2,32) = 0.46, *p* = 0.63, η_p_^2^ = 2.8, observed power = 0.12). Planned comparisons between verb types across different motion conditions revealed that causatives yielded faster SOTs than perception/psychological constructions only in the toward condition for items analysis (*t2*(16) = 1.80, *p* = 0.047), but not for subjects. No other comparisons between verb types across different levels of the motion variable reached significance. The magnitude of SOTs in the toward condition was similar to those obtained in studies that employed a similar methodology but with static pictures/drawings of scenes, such as Kamide et al. (2003), although larger than those of Altmann and Kamide (1999). The lack of a verb effect in the away and neutral conditions is surprising. Allied to the motion condition main effect, the lack of verb effect in the away and neutral conditions suggests that eye-movements are controlled primarily by the agent motion, with saccades to target objects in the away and neutral conditions showing *insensitivity* to verb-thematic properties. In addition, there were no *anticipatory* effects, since saccades to the target object occurred 140 ms *after* the noun offset in the fastest causative-toward condition (in the slowest case, the perception-neutral condition, the SOT to the target occurred 678 ms after the noun offset). Thus, even in the fastest condition, with constraints posed both by the action of the agent (moving toward a given object as opposed to moving away from the scene or remaining neutral) and by the highly constraining causative verb, programming of the saccade may have occurred within the noun object. This interpretation takes into account the best estimate of a 200 ms attentional shift preceding the saccade, as made in other static-scene visual-world studies.

For the LME analyses, SOT to target was entered as the dependent variable, motion type and verb type were entered as fixed effects, and participants and items as random predictors. The SOT model was compared to a null model consisting of only random predictors and was found to provide a better fit to the data, χ^2^(5) = 84.83, *p* < 0.001. We also found a main effect of motion type, but no main effect of verb type or interaction (see Table 1).

**TABLE 1:** Linear regression of Saccade Onset Time.

**Predictor**	**β**	**SE β**	***t*-value**	**95% CI of β**	**Null comparison**
Constant	1402.64	125.90	11.14	[934.08, 1923.87]	
Motion type	–136.67	35.71	–3.83	[−368.85, 69.94]	χ^ 2^(2) = 29.73, *p* < 0.001
Verb type	79.86	57.92	1.38	[−249.01, 373.07]	χ^ 2^(1) = 1.67, *p* = 0.197
Motion type × Verb type	8.605	71.23	0.12	[−131.01, 148.22]	χ^ 2^(2) = 0.08, *p* = 0.961

Multiple comparisons revealed that there was a significant difference in SOTs between the toward and away motion type conditions, *p* = 0.001, and the toward and neutral conditions, *p* < 0.001, but no difference between the away and neutral conditions, *p* = 0.264, suggesting that participants are faster to look at the target object when the agent was in the toward condition. Furthermore, planned comparisons revealed no statistically significant differences between causative and perception verb types on all three levels of motion type (all *p* = 0.79), in contrast with the pairwise analyses we performed employing *t1* and *t2* separately. We thus suggest that there is a weak, though non-anticipatory effect of verb type (numerically faster causatives) in the toward condition.

### Number of Saccades to Reach the Target Object

**FIGURE 5:**
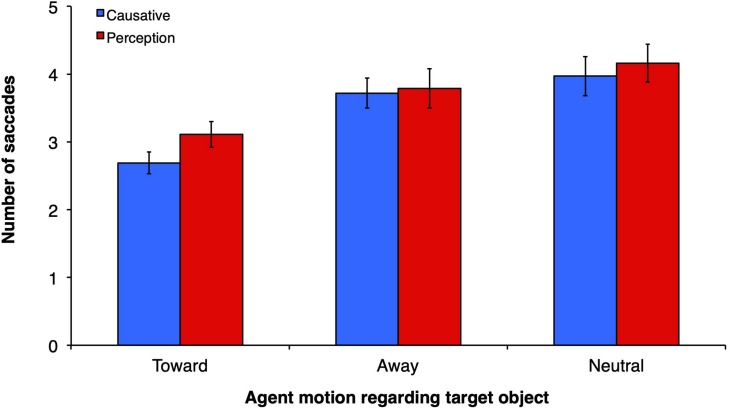
Mean number of saccades from the onset of the verb until the time participants fixate on the target object (grammatical complement of verb).

For number of saccades (Figure 5), a similar pattern of results was obtained: main effect of motion type (*F1*(2, 60) = 9.07, *p* < 0.0001, η_p_^2^ = 23.2, observed power = 0.969; *F2*(2, 32) = 5.88, *p* = 0.007, η_p_^2^ = 26.9, observed power = 0.842) but no main effect of verb type (*F1*(1, 30) = 0.37, *p* = 0.55, η_p_^2^ = 1.2, observed power = 0.091; *F2*(1, 16) = 2.12, *p* = 0.17, η_p_^2^ = 11.7, observed power = 0.278). There was a significant interaction between verb type and motion type (*F1*(2,60) = 7.81, *p* = 0.001, η_p_^2^ = 20.7, observed power = 0.942, *F2*(2,32) = 0.247, *p* = 0.78, η_p_^2^ = 1.5, observed power = 0.09). The number of saccades within the scene until the subject reached the target object for the first time, counting from verb onset, was relatively small–from 2.69 in the causative-toward condition to 4.2 in the perception-neutral condition. In the present study, the number of saccades also suggests that fixations were fast (in the magnitude of 298 ms each for the faster causative-toward condition). Planned comparisons revealed that in the toward motion condition the number of saccades to the target object was smaller when the verbs were causatives than when they were perception/psychological (*t1*(30) = 5.23, *p* < 0.0001; *t2*(16) = 2.62, *p* = 0.011). No other comparisons between verb types across motion conditions were significant.

For the LME analyses we used number of saccades from verb onset until object reached the target as the dependent variable. Motion type and verb type were entered as fixed effects, and participants and items as random predictors. This model was compared to a null model consisting of only random predictors and was found to provide a better fit to the data, χ^2^(5) = 35.54, *p* < 0.001. Similar to our SOT LME analyses, we found a main effect of motion type, but no main effect of verb type or interaction (see Table 2).

**TABLE 2:** Linear regression for total number of saccades before reaching target object.

**Predictor**	**β**	**SE β**	***t*-value**	**95% CI of β**	**Null comparison**
Constant	2.73	0.53	5.19	[3.14, 6.18]	
Motion type	0.58	0.10	6.07	[−1.34, −0.11]	χ^ 2^(2) = 34.89, *p* < 0.001
Verb type	−0.22	0.29	−0.77	[−1.10, 0.87]	χ^ 2^(1) = 0.68, *p* = 0.411
Motion type × Verb type	0.12	0.19	0.62	[−0.21, 0.57]	χ^ 2^(1) = 0.39, *p* = 0.534

Multiple comparisons indicated that there was a significant difference in number of saccades between the toward and away motion type conditions, *p* < 0.001, the toward and neutral conditions, *p* < 0.001, but no statistically significant difference between the away and neutral conditions, *p* = 0.35, indicating that participants performed less saccades before reaching the target object when the agent moved toward that object. Planned comparisons revealed no statistically significant differences between the causative and perception verb types on all three levels of motion types (all *p* = 0.96). Numerically, however, the number of saccades to reach the object in the causative condition was smaller (2.69) than in the perception condition (3.11). The magnitude of these values is similar to those obtained by Staub et al. (2012), in their restricting (2.77) and control (3.23) conditions.

### Cumulative Saccades to the Target Object

Our time-course analyses relied on cumulative saccades to the target object, rather than the usually reported proportion of fixations per trial. As we have seen in the review of visual world studies, proportion of saccades divided by trials and time slots tend to produce significant effects–thus reported as “anticipatory”–even when a small proportion of looks to target is obtained. For instance, in the Kamide et al. (2003) study, the contrast between the two conditions was significant even when only 7 and 10% of the saccades have been made to the target, thus, during time slots at which 93 and 90% of all other saccades were directed elsewhere in the ersatz scenes. Notice also that because the scenes were impoverished and contained only about four to six potential targets (with a smaller number of potential targets at every new target fixated), reported effects of anticipatory eye movements often occur with proportions that are below chance, even if we were to assume that all objects are equipotential in terms of salience.

**FIGURE 6:**
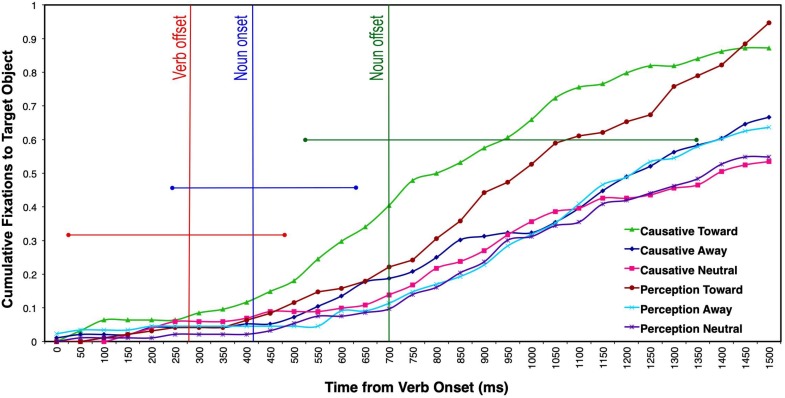
Cumulative number of saccades launched to the target object after verb onset as a function of different motion and verb conditions. The vertical bars represent the mean verb offset (red), noun onset (blue), and noun offset (green) for the two linguistic conditions. The horizontal straight lines represent the length range of each of the three words.

The ANOVAs and LME analyses took into account 31 bins of 50 ms each (up to 1550 ms), which corresponded to the maximum length of the noun complement. For the analysis of cumulative saccades (see Figure 6), there was a main effect of motion type (*F1*(2, 60) = 59.85, *p* < 0.0001, η_p_^2^ = 66.6, observed power = 1); *F2*(2, 32) = 4.49, *p* = 0.019, η_p_^2^ = 21.9, observed power = 0.727), a main effect of verb type only for participants (*F1*(1, 30) = 46.62, *p* < 0.0001, η_p_^2^ = 60.8, observed power = 1; *F2*(1, 16) = 0.01, *p* = 0.932, η_p_^2^ = 0.01, observed power = 0.051), and a significant verb × motion interaction by participants only (*F1*(2, 60) = 35.29, *p* < 0.0001, η_p_^2^ = 54, observed power = 1; *F2*(2, 32) = 0.41, *p* = 0.67, η_p_^2^ = 2.5, observed power = 0.11). The only difference between these analyses and the previous types of analyses (SOT and number of fixations) was that of a main effect of verb type, as well as an interaction between verb type and motion type (by participants), mostly because cumulative fixations take into account verb effects over time, when fixations to the target object are close to 1 for the two toward conditions. Planned comparisons revealed that in the toward motion condition, the cumulative number of saccades to the target object was greater when the verbs were causatives than when they were perception/psychological, in the participants’ analysis (*t1*(30) = 7.26, *p* < 0.0001; *t2*(16) = 0.77, *p* = *0.45*). No other comparisons between verb types across motion conditions were significant. It is important to highlight the main finding stemming from this analysis: The number of saccades to the target occurring at noun offset (750 ms bin from verb onset) is relatively small (*M* = 0.4), even in the fastest causative-toward condition. It appears that this condition shows an earlier peak than the other conditions during the processing of the noun, although the difference is only significant when noun offset is considered in the analysis. This effect suggests that this condition is set apart early on, although–as in the SOT analysis–this is not indicative of an anticipatory effect to target.

For the LME analyses, motion type, verb type, and bin were entered as fixed effects, and participants and items as random predictors. This model was compared to a null model consisting of only random predictors and was found to provide a better fit to the data, χ^2^(7) = 278.73, *p* < 0.001. We found a main effect of motion type and bin, a significant interaction between motion type and verb type, as well as a marginally significant interaction between motion type and bin (see Table 3).

**TABLE 3:** Linear regression of cumulative number of saccades.

**Predictor**	**β**	**SE β**	***t*-value**	**95% CI of β**	**Null comparison**
Constant	8.88	1.49	5.97	[0.60, 1.18]	
Motion type	1.56	4.99	3.12	[0.06, 0.25]	χ^ 2^(1) = 5.58, *p* = 0.018
Verb type	7.35	8.34	0.88	[−0.09, −0.24]	χ^ 2^(1) = 0.76, *p* = 0.379
Bin	1.61	6.57	2.45	[0.00, 0.03]	χ^ 2^(1) = 5.99, *p* = 0.014
Motion type × Verb type	−8.67	2.56	−3.40	[−0.14, −0.04]	χ^ 2^(1) = 11.53, *p* < 0.001
Motion type × Bin	3.52	1.97	1.79	[0.003, 0.007]	χ^ 2^(1) = 3.21, *p* < 0.073
Verb type × Bin	2.75	3.24	0.85	[0.004, 0.009]	χ^ 2^(1) = 0.72, *p* = 0.395
Motion type × Verb × Bin	−2.54	−3.94	−0.64	[−0.01, 0.01]	χ^ 2^(1) = 0.41, *p* = 0.520

Planned comparisons revealed that there was a significant difference in the cumulative number of saccades to the target object between the causative toward and perception toward condition, *p* = 0.006, suggesting that participants made a greater number of saccades to the target object over time when the verbs were causatives and when the agent was moving toward the target object. Similarly, results also showed a significant difference between the toward and away motion type conditions, *p* < 0.001, the toward and neutral conditions, *p* = 0.007, but no statistically significant difference between the away and neutral conditions, *p* = 0.999.

## General Discussion

The present study addressed two fundamental questions on the relationship between linguistic and visual integration. The first was the nature of this integration, here operationalized as saccades to objects in a dynamic scene as a function of verb onset in the speech stream. The second was the architecture of the visual-linguistic interaction that affords our ability to “talk about what we see” as Macnamara (1978) put it. This second question bears on the nature of the representations that both systems compute which enables them to “talk” to each other. We assumed–following Jackendoff and others (e.g., Fodor, 1975; Jackendoff, 1987, 2012) – that the interaction between language and vision requires amodal, rather than modality-specific codes. We begin our general discussion by addressing the effects we obtained with dynamic scenes and contrasting them with those obtained in other, static-scene visual world studies. We follow this with a discussion of the model we presented schematically in the Introduction, addressing in particular the nature of the interaction between language and vision and the representations they might deploy.

### The Present Findings and Contrast With Static Visual World Studies

We made two main predictions. First, consistent with an interactive view of visual-linguistic architecture, we predicted that we would obtain faster saccades in the case of the more restrictive causative verb, than in the case of the perception/psychological verb. This prediction was consistent with those made in other studies (e.g., Altmann and Kamide, 1999; Staub et al., 2012) manipulating static scenes. Second, we predicted an interaction between verb type and context such that causatives would lead to faster saccades across all motion conditions. Moreover, we predicted anticipatory eye movements in the case of causatives which would signal an early interaction between verb meaning and scene. In contrast with these predictions, a lack of verb-type effect across motion conditions, and a lack of *anticipatory* verb effects would signal that viewers/listeners process linguistic and visual information independent of each other during initial stages, with a late interaction between the two systems.

Our findings seem to show a lack of early interaction. We obtained agent motion effects and a weak effect of verb type in the toward motion condition only, with causative verbs yielding marginally faster saccades to the target than perception verbs in the items analyses, as well as smaller number of saccades, and in the cumulative saccades over time. Moreover, we found no anticipatory effects in any of the conditions. Thus, hearing *crack* in a cooking context does not lead to anticipatory saccades to a highly related object *egg*, not even when the agent moves toward the object. This might suggest that background information computed from the scene (viz., the encoding of objects that populate a scene), or expectations about the unfolding event, do not influence initial verb-complement processing. In fact, it appears that eye movement processes are independent of linguistic interpretation when the scene is dynamic. That is, in dynamic scenes, attentional processes seem to lock onto relevant properties of the visual event–such as the *Agent* activity–without being influenced by properties of the linguistic stream, in particular by the selection of verb-complement *Theme* referents. This suggestion, however, should be seen with caution given that the lack of interaction between verb and motion type in the SOT analyses yielded low power.

We found that most of the saccades and brief fixations during the initial processing of the event were to the region of the agent (on average, this was a time window of 749 ms, going from the acoustic onset of the verb to the acoustic offset of the noun complement of the verb). These observations are in part consistent with previous studies on static scene processing without linguistic stimuli, which have found that focal attention is primarily directed toward human figures (Henderson and Ferreira, 2004; see also Yarbus, 1967, Ch. 7). This is also consistent with findings by Kamide et al. (2003) and Boland (2005) who showed that most post-verbal fixations were to the animate agents in the scene.

Our results appear to be at odds with previous studies that showed influence of visual/background context on linguistic processing and, in particular, they are at odds with results suggesting that eye movements to static scenes are locked into ongoing linguistic processes of verb-thematic assignment. We have in fact questioned those results, in our review, because they are often based on a small difference between conditions, or because they rely on a small proportion of fixations, or occur relatively late, post verb-complement offset. In our analyses of cumulative saccades, we do find an effect of verb type, with causatives leading to more saccades to target objects over time; however, this effect only occurs between verb offset and noun offset. But it is important to note that a significant effect of verb type here cannot be indicative of an anticipatory effect. Notice that data at that segment represent only an average of one tenth of a saccade. As we pointed out in our review of the literature, we could not argue that there is an anticipatory effect with such a small proportion of data. Thus, although it is possible that an effect of verb restriction begins to appear early on during verb processing, the effect is restricted by a very limited amount of data, and only when one does not take latencies or number of fixations into account.

The small verb type effect found in the toward condition may be better explained as a late, *confirmatory* effect, that is, a late integration between verb and scene information. This is so because eye movements are triggered by post-verbal information, and occur only when agent motion is indicative of which object the agent is about to interact with. Recall that agent motion disambiguation (toward or away from target object) is synchronized with verb onset in the sentence and spans a window of time of about 234 ms (7 frames). The onset of agent motion may be rapidly combined with verb semantics, but surprisingly not before the noun information is available. When the agent is moving toward a particular object, eye movements are drawn to that location, with computed verb-thematic information being used to further *confirm* the potential saccade-landing site.

It is possible, however, to take this confirmatory effect as representing a true interaction between agent-directed motion and verb-*thematic* (thus, by hypothesis, *linguistic*) information. In our contrast between verb classes, we hypothesized that the verb’s thematic properties, which for causative verbs require a particular object to undergo a change of state (a *Patient* or *Theme*; see Levin and Rappaport Hovav, 2005), would draw attention to a potential *Theme* in the event. We predicted that the same effect would not be found in the case of the perception/psychological class because, contrary to causatives, the *Experiencers* (“agents”) of perception/psychological verbs are the very entities that supposedly undergo a “change of state” (or that experience an object). Dissociations between agentives and *Experiencer* verbs have been found in Alzheimer’s and aphasia patients (Piñango, 2006; Manouilidou et al., 2009), and the relative difficulty of *Experiencer* verbs has been attributed to their non-canonical thematic structure (no *Agent* role). Thus, the predicted difference between verbs in these two conditions (*Theme* that undergoes a change of state by the *Agent*; and *Theme* that causes a change of state in the *Experiencer*) should have contributed to *enhance* the differences in eye-movement behavior, if thematic roles were constraining referents in the visual context. But this was not the case in two of the motion conditions. Even if what we observed in the causative-toward condition, then, is an effect of typically *linguistic* representations interacting with information computed about the scene, they represent late effects. As we will discuss, in the context of the model presented in the next section, the conceptual representation of verbs might rapidly interact with conceptual representation of scene information to direct gaze to objects.

A key component of our investigation is the use of dynamic scenes and the manipulation of visual/motion context. But this manipulation introduces variables that may hinder a direct comparison with studies employing still pictures and ersatz scenes. For instance, dynamic scenes might be more cognitively taxing, obfuscating an otherwise early interaction between visual context and linguistic processing. Andersson et al. (2011) demonstrated that when presented with highly complex static scenes coupled with fast-paced sentences containing four nouns referring to objects in the scenes, participants have difficulty keeping track of referents, yielding saccade latencies in the magnitude of 2500 ms post-noun onset. Our scenes, however, are not “hoarding” scenes as those employed by Andersson et al. (2011), our sentences were recorded at a normal pace, and referred primarily to only one object in the scene (internal argument of the verb), other than the agent, in the relevant contrast. In addition, as we discussed above, our verb contrasts and the toward motion condition should have contributed to *enhance* anticipatory effects.

It is also possible that agent motion in the scene grabs overt attention, even when the verb might be covertly directing attention to its related object–thus reflecting an early interaction that is not manifested in early saccades. We have no direct evidence that subjects might be withholding a saccade in spite of an attention switch to the target object. But we contend that fixations on moving agents were not simply a function of low-level motion because fixations remained on the agents for up to 2 s after the onset of motion, suggesting that attention to agents is mostly goal-directed instead of stimulus-driven (Hillstrom and Yantis, 1994). In addition, results from our laboratory, in which we employed a change blindness task with dissolving objects during realistic dynamic scenes, demonstrate a similar effect: the sudden motion of the dissolving target object did not capture attention, which is initially directed to the agent of the event (van de Velde, 2008). Related inattention blindness effects in dynamic scenes, but with human targets, have been reported (Simon and Chabris, 1999). Thus, it appears that the effects we obtained in the present study cannot be simply attributed to motion in the scene preventing attention to verb-related objects. And even if we were to attribute delayed saccades to the object due to agent motion, a typical linguistic by visual processes interaction would allow for verb effects to be obtained *consistently* within scene types, which was not the case in the present experiment.

As observed by a reviewer, one other factor may have potentially delayed saccades to targets contributing to our lack of anticipatory effects, even in the most constraining causative-toward condition: the post-experimental recall task, which could have promoted memorization rather than rapid comprehension of sentences and scenes. We contend, however, that our task promotes comprehension (scene gist, sentence proposition), which is what participants later use to recognize material in the post-experiment task. Other studies have encouraged subjects to look at objects (e.g., in Andersson et al., 2011) with questions at every trial. Ito et al. (2018), requested subjects to keep a list of words in mind while performing the visual world task (a cognitive load manipulation) and obtained different effects compared to a no-load condition. However, our post-experimental task is a simple cued recall task of sentences and scenes similar to asking subjects questions after a trial. We reiterate that in our causative-toward condition subjects have both verb and motion information to anticipate the target object.

On the flip side, anticipatory effects may occur primarily in static and ersatz scenes–thus they might not be the *norm* of language use across visual contexts. It is possible that in situations where the scene is static (for instance, dishes on a dinner table or objects on a shelf) the viewer/listener might be more sensitive to linguistic information, even anticipating the nature of referents.^8^ The same applies to goal-directed tasks (e.g., Tanenhaus et al., 1995). It is to these situations that the studies we reviewed best apply. But their use to generalize about language use in context tout court can be challenged by the present results.

In summary, the results of our experiment suggest that visual attention and linguistic processing may be computed independently and in parallel, in the construction of *dynamic* event representations. It seems that the processing of dynamic naturalistic scenes and sentences occurs without visual attention being initially influenced by the nature of the linguistic stream, and without representations built from visual context influencing the early selection of linguistic referents *by necessity*. This apparent decoupling of linguistic and visual processes during the early moments of linguistic and visual-scene perception may be one further indication that the two systems are modular, rather than interactive, with interaction occurring at a later stage.

### *Where* and *How* Do (the Products of) Vision and Language Interact?

A second more fundamental question that the present article addresses is the nature of the architecture of the visual-linguistic system and how their respective representations are combined. There seems to be little doubt in cognitive science that information computed by visual and linguistic systems *should be* integrated at some processing level and that this integration needs to rely on a common representational code (Fodor, 1975, 2008; Macnamara, 1978; Jackendoff, 1987, 2012). The assumption of a common representational code is not exclusive of symbolic approaches to cognition, as in the cited works, but a characteristic of highly interactive models as well (McClelland et al., 2014). The model we propose, however, takes linguistic and visual processes to share information at a higher-level conceptual system, with their early inputs being encapsulated.

A modular encapsulated system in a symbolic cognitive architecture is sensitive to *formal* properties of the information it computes (Fodor, 2000). In our model, these formal properties include, among others, word and sentence structure and argument/thematic properties of verbs, on the linguistic input, and feature combination, scene layout, and token object discrimination, in the visual input. These input systems are tuned to *different natural kinds*, and they appear to operate on different formal properties but to produce outputs that might serve the interpretation of events relying on common predicates.

**FIGURE 7:**
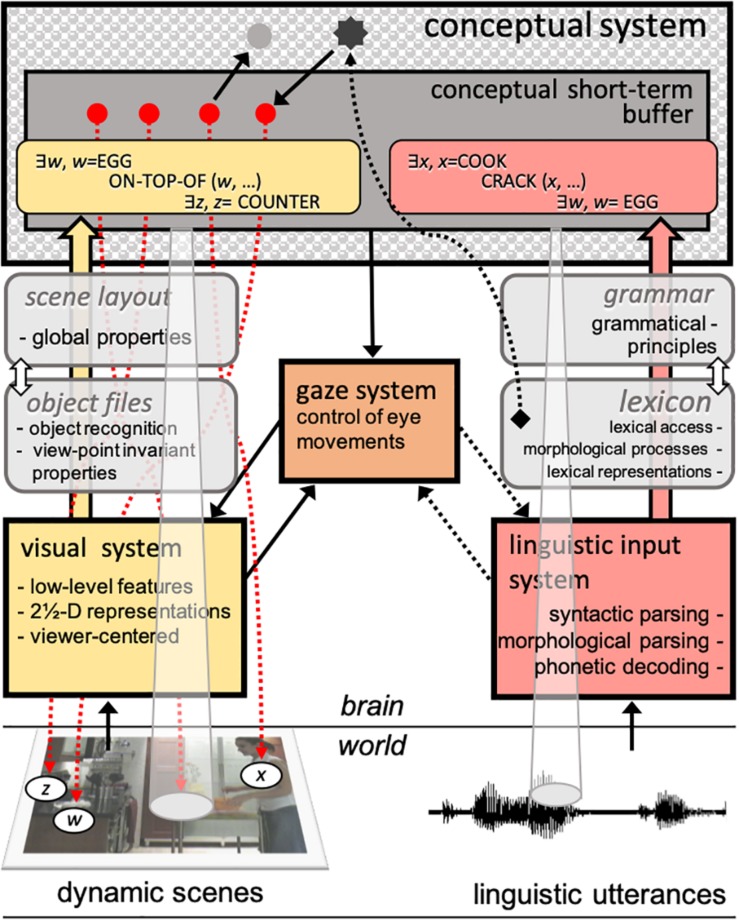
The workings of the modular dynamic visual-linguistic interaction model. See Supplementary Figure S2 for detailed description.

One of the most promising attempts to conceive these common predicates that serve the interaction between linguistic and visual representations has been that of Jackendoff (see, in particular, Jackendoff, 1987, 2012). The model we present in Figure 7 proposes a similar view of the visual and linguistic systems (For a more detailed description of the workings of the model, see Supplementary Figure S2).

The model takes the early inspection of a scene concomitantly with the analysis of linguistic input to be computed independently and in parallel by the two systems. These two systems bind scene and sentence constituents into predicate-like structures. More specifically, relations between objects in the scene and unfolding events are represented in a language that is common to the *output* of both visual and linguistic systems. In the example in Figure 7, the computation of propositions is couched in a more neutral predicate-logic notation to exemplify how representations of objects and their relations obtained from the unfolding linguistic and visual domains might be combined. In Jackendoff’s (1987, 1990) more detailed notation, conceptual structures related to *States* and *Events* (as well as other ontological categories such as *Thing* and *Place*) might be continuously computed from scene information, thus forming a framework for the interpretation of unfolding linguistic messages. These conceptual-structure representations computed from an event such as the one presented in Figure 1 may consist of expressions such as those in (1).

(1)
  (a.) [_State_ BE ([_Thing_ WOMAN], [_Place_ IN ([_Thing_ KITCHEN])])]
  (b.) [_State_ BE ([_Thing_ EGG], [_Place_ ON ([_Thing_ COUNTER])])]
  (c.) [_Event_ PUT ([_Thing_ WOMAN ([_Event_ FLOUR, [_Place_ IN ([_Thing_ BOWL])])])])]
  (d.) [_Event_ GO [_Thing_ WOMAN [_Path_LEFT]]])^9^

This proposal is also compatible with the idea that dynamic scene inspection and action require the binding and tracking of multiple elements within the “perceptual circle.” These elements are represented as visual predicates computed early on about indexed (or FINSTed) scene constituents (see, e.g., Fodor and Pylyshyn, 2015; Pylyshyn, 2018).

Besides being explicit about the types of representations that might constitute the interaction between linguistic and visual processes, an advantage of the present proposal over connectionist models mentioned above, is that the codes that constitute conceptual structure (and in particular those temporarily placed in the CSTB) are computable; that is, the unfolding processes are carried over as a function of the formal properties of the expressions. The expressions are also compositional–so that the propositions that they express are indeed a function of their constituent elements and their structure. Simply put, connectionist networks need to account for how the nodes that stand for, say, WOMAN, CRACK, COUNTER, and EGGS, bear the relation that they do such that the sentence means that the woman will crack the eggs that are on the counter. A similar point can be made about the concepts computed from the scene: they need to be bound into events. Without postulating *structural relations* between the “activated” elements, there is no saying on what they mean together (see Fodor and Pylyshyn, 1988).

Sentence processing during dynamic events occurs in contexts that thus might be rich in conceptual representations computed *from* scene inspection–with sentences contributing to attention focusing on (and “indexing” of) what is happening in the scene. In the case of the study reported here, attention is directed toward the interaction between *Agent* and affected object or between *Agent/Experiencer* and the causer of psychological state (both referents of *Themes* in the sentences). As can be seen in Figure 7, we assume that the semantic representation of the sentence also takes these same conceptual-structure codes, thus contributing content to the ongoing conceptual representation of the event that is seen and heard concomitantly. In sum, the interaction between predicate structures computed from scene and sentence representations are computed in parallel and integrated in real time based on the products of their respective input systems.

It is difficult to say whether the effects we obtained are modular in the now classical sense (Fodor, 1983) or whether they represent a type of encapsulation typical of even higher cognitive systems (Barrett and Kurzban, 2006; Chomsky, 2018). The classical type of modularity system encompasses only mandatory perceptual computations, which are domain-specific, and are not influenced by other input systems or by general cognitive processes such as those of long-term memory, expectations, and desires. The higher-cognitive type of modular system exhibits some of the properties of input modules, but is primarily defined by the type of knowledge domain it operates on–akin to Chomsky’s (2018) view of modules as central systems. Thus, even logical inferences might be *modular* if they are, as Barrett and Kurzban (2006) suggest, “construed in terms of the formal properties of information that render it processable by some computational procedure” (p. 634). In the present case, it is possible to assume that visual contextual information influences on language are “domain-specific” in this sense, if language-vision interactions are computed over a common code at a higher level than their classical input modules. It could be the case that vision and language have *evolved* to work together in action and communication, and thus, they might operate with representations from both classical input domains. If this is the case, then effects of context on parsing decisions (such as those reported by Tanenhaus et al., 1995; Spivey et al., 2002) or putative effects of context on thematic-role processing, as measured by eye-movement behavior, could be manifestations of a common domain of knowledge, a modular higher-cognitive system in the sense proposed by Barrett and Kurzban (2006) and Chomsky (2018). It is difficult to tease apart these proposals for modularity but they do constitute alternative interpretations for some now established effects of context in language and for the effects we found.

All studies employing the VWP with static scenes stress how fast the interaction between visual and linguistic representations might be, given the anticipatory or early post-verbal effects commonly found. Contrary to static scenes, dynamic events as the ones employed in the present study, rely on representations computed from both visual and linguistic inputs being continuously updated, that is, they need to allow for dynamic interaction of information in working memory. In our model, these computations are occurring at the CSTB (akin to Potter, 1999; see also Potter, 2018). The system accesses long-term memory representations of words and objects and builds conceptual structures compatible with both visual and linguistic predicates. We suggest, then, that the locus of the *effects* of visual contextual influence in sentence processing is post-perceptual, that is, when both visual and linguistic *outputs* have reached the conceptual buffer and expressions about the unfolding event are being built.

Given that access to conceptual information about objects and scenes may occur within 100–200 ms of scene onset (Potter and Faulconer, 1975; Thorpe et al., 1996; see also Supplementary Figure S2), it would be expected that information about the scene would guide the gaze to the appropriate referents as sentences unfold–and in particular, as verbs make certain objects in the naturalistic scenes potentially more prominent for further processing. If so, objects of causative sentences and their referents in the world would have an advantage over objects of perception verbs. Clearly, in our study, eye movements are controlled more by what the visual context “says” about the event than by what the sentence says, and very little by the interaction between the two systems. Although most research on scene gist processing has been done with static scenes (see Henderson and Hollingworth, 1999), eye movement studies on scene processing suggest that while the gist is obtained rapidly, consolidation of scene details continues and requires possibly indexing and serial visual routines to integrate information (Pylyshyn, 2003). The processing of the category or the gist of a scene may rely on determining the meaning or category of one of its constituent objects; the initial representation of objects and scene, however, need not be conceptual: Henderson and Hollingworth (1999) as well as Pylyshyn (2003, 2007) point to the structural or even “pre-semantic” nature of scene perception in vision. What happens after the initial analysis of the scene requires further computations–processes over conceptual-structure representations– which are likely to take into account sources of information such as the products of linguistic input. We take these high-level computations and the context effects that they engender to occur post-perceptually, relying on the structural analyses provided by both language and visual input systems.

## Conclusion

We have raised concerns about the generalizability of studies that support a fully interactive–rather than a modular–view of functional architecture using impoverished static rather than realistic and dynamic scenes. One of our primary concerns is that the claims made in support of an interactive–so called *incremental*–linguistic system rely on stimuli that do not necessarily represent the use of language in dynamic visual contexts. Stimulus variables such as scene complexity and motion call into question the conclusion that the linguistic system takes contextual information into consideration at a very early stage, with linguistic input analysis being sensitive to information available to the visual system (Tanenhaus et al., 1995). Given the potential usefulness of the visual-world paradigm for understanding how different perceptual and cognitive systems make their representations available to each other, it is important to consider all possible alternative *loci* of influence or interface between the representations computed by different input and cognitive systems.

We suggest, then, that the locus of the previously observed visual context effects on sentence processing may actually be *after* the initial analysis of linguistic tokens, where alternative sentence parses or interpretations may be selected for further processing or may be re-analyzed according with a particular visual context representation. In the present study, eye movements to referents of verb arguments occur after the *offset* of the noun complement (ranging from 140 ms in the causative-toward condition to 700 ms in the perception-neutral condition). At that point verb-thematic properties across contexts have been processed, well in advance of eye movements to verb-related objects. Visual context may help guide attention to objects in the scene only when the *interpretation* of the critical verb phrase is under way. Agents of dynamic events, however, appear to grab the focal attention of the viewer/hearer, thus making eye movements to what is seen initially insensitive to what is heard–in particular without the seemingly mandatory verb-structural and thematic effects found in studies with static scenes (e.g., Altmann and Kamide, 1999; Knoeferle et al., 2005; Staub et al., 2012). In our study, “what was heard” was not ignored, as participants were able to interpret/encode the sentences despite not tracking word-referents on the screen continuously (see also Andersson et al., 2011) and without being sensitive to verb-thematic properties in two of the motion conditions. That is, sentences and scenes were independently processed and information about both was integrated at post-perceptual stages; in our proposal, the post-perceptual interaction between the two input systems occurs at a conceptual buffer and takes the form of common-code conceptual, predicate-like structures.

In conclusion, what is striking about the effect we obtained–an insensitivity to verb distinctions when agents do not engage objects–is that it is commonly believed that our attention is usually tied to the products of our linguistic processes, in particular in visual contexts (Tanenhaus et al., 1995; Altmann and Kamide, 1999; Spivey et al., 2002). The popularity of the visual world technique (see Huettig et al., 2011) attests to its perceived usefulness to investigate linguistic processes and their interaction with visual context. But we question its effects insofar as impoverished scenes are employed. We found that the process of understanding a dynamic visual event and understanding a sentence describing the event appear to be largely independent of each other during the initial processing of visual and linguistic input. The caveat is that the two processes–visual and linguistic–interact with each other when, besides linguistic cues, agents disambiguate the nature of their actions. When this happens–for instance, when agents walk toward a given object–then verbs that are supposed to “select” for these objects trigger faster and a smaller number of saccades to these objects than neutral verbs do. However, even in these cases, when causative verbs seem to constrain the domain of reference to one object and when the agent reaches toward that object, saccades are *not* anticipatory, thus challenging results supporting interactive models of language processing. Further, from a methodological standpoint, it seems clear that the use of dynamic rather than static scenes constitutes a more ecologically valid method in the study of the potential influence of visual context on language comprehension, thus representing an advance in the investigation of the interaction (or lack thereof) between these key cognitive and perceptual systems. We believe we found support for the independence–or modularity–of linguistic and visual processes employing a task that better exemplifies realistic uses of language in dynamic scenes. We have proposed that the two systems interact only at a central, conceptual system that operates over the *outputs* of vision and language input systems, and relies on a common, propositional code. Advancing Macnamara (1978) quest for understanding how we talk about what we see (or how we understand what we hear and see concomitantly), we have proposed a model that takes visual and linguistic predicate-argument relations to be the basis of language use in visual contexts.

## Ethics Statement

This study was conducted in accordance with the recommendations of the Concordia University Human Research Ethics Committee and was approved by this committee. All subjects gave written informed consent in accordance with the Declaration of Helsinki.

## Author Contributions

RA is the main author of the manuscript, the principal responsible for the conception and design of the study, production of materials, theoretical background, and general discussion. JD conducted the experiment, and contributed to the statistical analyses and to the manuscript. CA performed the main statistical analyses and contributed to the final manuscript. MG contributed to the conception and design of the study, and to the production of materials.

## Conflict of Interest

The authors declare that the research was conducted in the absence of any commercial or financial relationships that could be construed as a potential conflict of interest.

## Footnotes

1 It should be noted that express saccades are obtained under very different conditions (e.g., after the onset of a dot in peripheral vision) and, to our knowledge, have not been investigated with more complex but static naturalistic stimuli such as those of Staub et al. (2012). Our suggestion, however, is that saccades to targets *could* occur at a latency that is close to the difference in length between the conditions in Staub et al.’s (2012) study.

2 We only briefly discuss–and hereafter refer to–Coco et al.’s (2016) Experiment 1 because their Experiment 2 employed a “blank screen” paradigm with a scene preview of 5000 ms, thus constituting a memory experiment.

3 Regarding (1), we deem this necessary to understand the reflexive (or lack thereof) saccades to target, which might signal the strength of the context by verb type interaction (see below, in Results, our analyses of this measure). Regarding (2) it is quite possible to obtain a difference in magnitude of proportion of fixations to the target with a very small number of fixations (see Kamide et al., 2003, for one example). Although this is not necessarily the case in Coco et al.’s (2016) study, to claim anticipatory effects from such data might lead to a generalization that stands on the exception rather than on what might be the rule.

4 It is important to note that the notion of interactive system usually proposed in the reviewed visual world studies assumes that modular systems allow for *no semantic* information guiding lower linguistic processes such as sentential parsing and word recognition. However, a recent proposal for incorporating semantics into the linguistic module (de Almeida and Lepore, 2018) would in part account for semantic effects in linguistic computations–viz., by regulating local discourse elements and possibly visually computed referents–to aid in the construction of a semantic representation, before other forms of knowledge (e.g., background/world knowledge) influence linguistic decisions.

5 See Jackendoff (2012) for details on how conceptual structure differs from other cognitive systems as those responsible for computing visual-spatial structure. See also Jackendoff (1990) on how concepts–the elements of conceptual structure–might be themselves structurally complex representations. In the present discussion, we gloss over the technical details of this proposal, in particular with regards to whether or not concepts are complex (see de Almeida and Manouilidou, 2015, and de Almeida and Antal, in press, for discussions on how concepts lexicalized by verbs might be represented).

6 A reviewer also pointed out that clauses with *Before*… have been found to affect working memory because, contrary to *After*… listeners/readers are required to alter the temporal order of events described in upcoming clauses, as suggested by an ERP study conducted by Münte et al. (1998). The assumption is that *Before* sentences could contribute to delaying saccades to target object. However, of our 17 sentences, only four began with *Before*, and three with *After*. See Supplementary Material for all sentences and scenes.

7 Notice that some of the visual world studies discussed above (e.g., Altmann and Kamide, 1999; Staub et al., 2012) included in their analyses trials in which participants were already looking at the target object at verb onset. They took the next fixation as “the first valid fixation on the target, as this is the first fixation that could, in principle, be guided by the selectional restrictions of the verb” (Staub et al., 2012, p. 932). We notice that this occurred on average in 21% of the trials in Staub et al.’s (2012) study, and in 10% of the trials in Altmann and Kamide’s study, compared to 6.4% in the present study.

8 We thank a reviewer for stressing this point.

9 In this example, we adopt a language-like representation following Jackendoff’s (1990) notation as a convenient way to represent the potential predicate structures computed from the event. But we do not assume that the predicates themselves are further decomposed, nor do we take the ontological categories–such as *Thing*, *Event*, etc.–to be necessarily the ones we encode from scenes or sentence meanings, although we see heuristic value in them.
