# Supplementary material for: Understanding Events by Eye and Ear: Agent and Verb Drive Non-anticipatory Eye Movements in Dynamic Scenes
Source: Front Psychol. 2019 Oct 10;10:2162. doi: 10.3389/fpsyg.2019.02162 (PMC6795699; doi:10.3389/fpsyg.2019.02162)
Supplement: Supplementary file 1 [file Data_Sheet_1.pdf]

Supplementary Material

Understanding Events by Eye and Ear: Agent and Verb Drive Non-Anticipatory Eye Movements in Dynamic Scenes

Roberto G. de Almeida[1*], Julia Di Nardo[1], Caitlyn Antal[1,2], Michael W. von Grünau[1]*

[1]Department of Psychology, Concordia University, Montreal, QC, Canada

[2]Department of Linguistics, Yale University, New Haven, CT, USA

*** Correspondence:**
Roberto G. de Almeida
roberto.dealmeida@concordia.ca

1 Supplementary Figure 1 (S1)

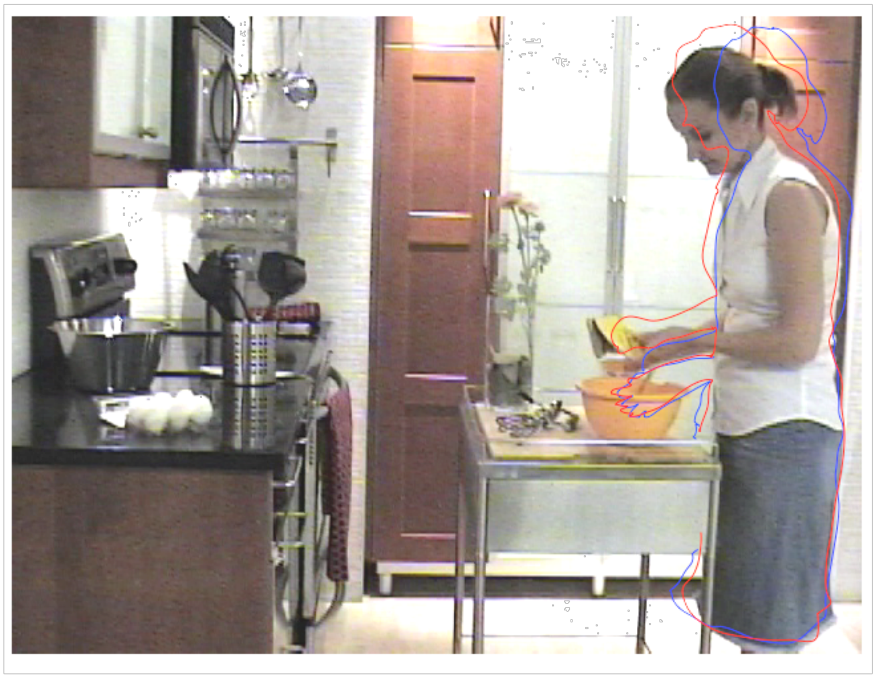

Supplementary Figure 1 (S1). Position of the agent in three motion conditions of the same event at verb onset. The frame is from a neutral motion condition film (i.e., when the agent—the cook—does not move toward or away from the target object—the eggs). The outlines represent the positions of the same agent in the corresponding frame of the movie in the toward motion condition (red), and in the away condition (blue). The scene and the outlines exemplify the different positions of the agent at the acoustic onset point of the two corresponding verbs (*crack* or *examine*), demonstrating the similarity between event onsets in the three versions of the movie. (Written informed consent was obtained from the depicted individual for the publication of this image).
