# Supplementary material for: Understanding Events by Eye and Ear: Agent and Verb Drive Non-anticipatory Eye Movements in Dynamic Scenes
Source: Front Psychol. 2019 Oct 10;10:2162. doi: 10.3389/fpsyg.2019.02162 (PMC6795699; doi:10.3389/fpsyg.2019.02162)
Supplement: Supplementary file 2 [file Data_Sheet_2.pdf]

Supplementary Material

Understanding Events by Eye and Ear: Agent and Verb Drive Non-Anticipatory Eye Movements in Dynamic Scenes

Roberto G. de Almeida[1*], Julia Di Nardo[1], Caitlyn Antal[1,2], Michael W. von Grünau[1]*

[1]Department of Psychology, Concordia University, Montreal, QC, Canada

[2]Department of Linguistics, Yale University, New Haven, CT, USA

*** Correspondence:**
Roberto G. de Almeida
roberto.dealmeida@concordia.ca

1 Supplementary Figure 2 (S2)

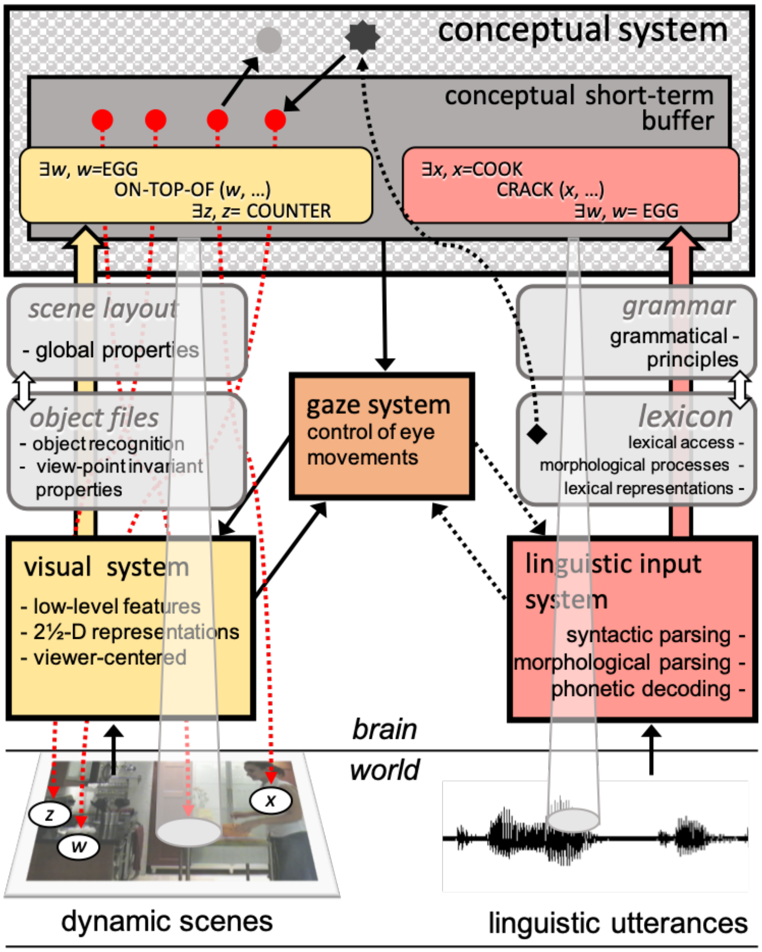

Supplementary Figure 2 (S2). *The workings of the modular dynamic visual-linguistic interaction model.* The two systems, linguistic and visual input, operate autonomously and in parallel with different algorithms, on different types of representations (their *natural kinds*) during the early stages of processing. The boxes exemplify subsystems and the types of representations and processes computed at different processing stages. The visual system produces its output initially based on the computation of low-level features also computed independently (Livingstone & Hubel, 1988), giving rise to a "2½-D" (Marr, 1982) type of representation that is viewer-centered. Recognition of objects in the scene rely on the combination of low-level features, stored properties of objects (possibly parts; Biederman, 1987) and global parsing principles (e.g., concave creases, vertices, surface and edge continuity; see also Hoffman & Richards, 1984). These objects are represented based on view-point invariant properties. Though disputed, based largely on response-time and neuroimaging data (see Peissig & Tarr, 2007), view-point invariant representations have the advantage of being productive, thus allowing for a limited number of basic representations to account for an infinite number of possible objects. Although the status of scene representations—as distinct from objects—is difficult to pinpoint in *nonsemantic* terms (see Henderson & Hollingworth, 1999), the proposal is that a special set of algorithms compute global properties of scenes such as the distinction between foreground and background, spatial layout, and properties of the 3D space such as relative distance between objects. The object and scene subsystems interact such that objects might be recognized faster in their original contexts (e.g., a blender in a kitchen, a fire hydrant at a street corner; see, e.g., Biederman, 1972). The model assumes that the flow of visual information is not semantically guided, with scene "gist" being accessed rapidly at the conceptual short-term buffer (CSTB). The output of vision is "descriptive", rather than depictive or imagistic. A key argument for taking objects and scenes not to be properly *visual* but *descriptive* representations is that images cannot be primary mental representations, nor are they computable. Images need to be "tagged" or coded to be interpreted (Pylyshyn, 1973). The proposal is that visual descriptions are produced as a series of proposition-like representations about what is seen and attended to. As presented schematically in the model, the visual stream dynamically updates accessed representations—their properties ('what') and their spatial locations ('where')—based on computations from both, object files and scene layout. The predicate-argument relations are built dynamically and rely primarily on the multiple objects that are grounded (FINSTed; see Pylyshyn, 2018, for a review of FINST theory) in the environment. The FINST mechanism is represented by red arrows 'locking' concepts to a limited set of referents {*x*, *y*, *z*, *w*}, in the scene. Notice that one of the main tenets of the FINST theory is that the token objects are individuated at first based on their more primitive properties (say, "*thing*(*x*)"), thus they are "nonconceptual". Numerous experiments relying on the multiple-object tracking technique show that several objects might be FINSTed simultaneously and that attention to these elements is independent of their properties (see Pylyshyn, 2018). This relation is simplified in the figure, for nonconceptual individuation is prior to access to concepts in the conceptual system. It is estimated that visual processes–from early vision to object and scene recognition—operate on a latency of up to 300 ms with scene and objects being recognized as early as 100ms (Potter, 2018; Rousselet, Joubert, & Fabre-Thorpe, 2005; see also Contini, Wardle, & Carlson, 2017). Because the system is dynamic, the hypothetical latency should be seen as a loop beginning with early features and building conceptual structural representations, with new elements being FINSTed continuously in complex events. The semantic properties or "gist" of the scene are thus seen as the conceptual representation of the early encoding of scene properties. In the linguistic input stream, parallel computations begin with the decoding of phonetic information (when input is spoken language) and proceeds with syntactic parsing and lexical computations (morphological parsing, lexical access) based on stored lexical information. Lexical access entails the tokening of concepts in the conceptual system. The grammar,

as depicted in the model does not serve the input system only, but the output system as well, and thus it may serve as a “central module” (Chomsky, 2018). It is not clear the degree at which syntactic and morphological parsing rely on core grammatical principles, for they may operate on principles that are parsing-specific or a combination of both (see Frazier, 1988). For instance, while relative clause structure is determined by core grammatical principles, preference for relative clause attachment is not a grammatical principle but one that might be specific to the early parsing system. Morphological processes involved in word recognition—and later lexical access—might also rely on parsing-specific strategies (see de Almeida & Libben, 2005). The output of the linguistic input system, much like the vision system, is a proposition-like language. And, as is vision, it is updated dynamically relying on compositionality principles such as predicate-argument saturation, coreference assignment, and ellipsis. Estimates of the timing of word recognition and lexical access vary widely, with visual priming effects, for instance, being robust with about 60 ms exposure of the prime (Forster, 1999), suggesting that word recognition processes may begin at that early stage but may last approximately 400 ms—depending on word length—when search for phonetic competitors might be under way (Allopenna, Magnuson, & Tanenhaus, 1998). Relevant to our model, this suggests that lexical access may take place concomitantly with object/referent recognition. Concepts are dynamically accessed either by vision or by language and “placed” temporarily in the CSTB, which computes the predicate-argument relations based on both types of input information, thus describing events based on what is seen and heard simultaneously. Eye movements can be triggered by the three major systems. From vision, by exogenous factors such as feature changes and sudden appearance of potential targets. At the same level, eye movements can be controlled by the linguistic input in reading processes (for saccades are, to a large extent, dependent on lexical factors; see Reichle et al., 2003). But eye movements are also controlled by endogenous factors, such as by information at the CSTB or in the control of actions (Land, 2009). It is by the combination of visual and linguistic propositions in tasks that involve both systems that effects of integration may be obtained. Both systems also rely on attention “spotlights” which, in the case of the visual system, keeps track of unfolding visual events (e.g., picking up spatial information about objects, selecting eye-movement targets) and, in the linguistic system, monitors both, the external source of speech and internal parsing mechanisms.

References

Allopenna, P. D., Magnuson, J. S., & Tanenhaus, M. K. (1998). Tracking the time course of spoken word recognition using eye movements: Evidence for continuous mapping models. *Journal of Memory and Language, 38*, 419-439.

Biederman, I. (1987). Recognition-by-components: A theory of human image understanding. *Psychological Review, 9*, 115-147.

Chomsky, N. (2018). Two notions of modularity. In R. G. de Almeida & L. R. Gleitman (eds.). *On Concepts, Modules, and Language: Cognitive Science at its Core* (pp. 25-40). New York, NY: Oxford University Press.

Contini, E. W., Wardle, S. G., & Carlson, T. A. (2017). Decoding the time-course of object recognition in the human brain: From visual features to categorical decisions. *Neuropsychologia, 105,* 165-176.

de Almeida, R. G., & Libben, G. (2005). Changing morphological structures: The effect of sentence context on the interpretation of structurally ambiguous English trimorphemic words. *Language and Cognitive Processes, 20*, 373-394.

Forster, K. I. (1999). The microgenesis of priming effects in lexical access. *Brain and Language, 68*, 5-15.

Frazier, L. (1988). Grammar and language processing. In F. J. Newmeyer (Ed.), *Linguistics: The Cambridge survey, Vol. 2. Linguistic theory: Extensions and implications*, 15-34. New York, NY, US: Cambridge University Press.

Henderson J. M., & Hollingworth A. (1999). High-level scene perception. *Annual Review of Psychology*, *50*, 243-271.

Hoffman, D. D., & Richards, W. A. (1984). Parts of recognition. *Cognition, 18*, 65-96.

Land, M. F. (2009). Vision, eye movements, and natural behavior. *Visual Neuroscience, 26*, 51-62.

Livingstone, M., & Hubel, D. (1988). Segregation of form, color, movement, and depth: Anatomy, physiology, and perception. *Science, 240*, 740-749.

Marr, D. (1982). *Vision: A* Computational *Investigation into the Human Representation and Processing of Visual Information.* San Francisco, CA: Freeman.

Peissig, J. J., & Tarr, M. J. (2007). Visual object recognition: Do we know more now than we did 20 years ago? *Annual Review of Psychology, 58,* 75-96.

Potter, M. C. (2018). The immediacy of conceptual processing. In R. G. de Almeida & L. R. Gleitman (Eds.), *On concepts, modules, and language: Cognitive science at its core* (pp. 239-248). New York, NY: Oxford University Press.

Pylyshyn, Z. W. (1973) What the mind's eye tells the mind's brain: A critique of mental imagery. *Psychological Bulletin*, *80*(1), 1-24.

Pylyshyn, Z. W. (2018). Scientific theories and Fodorian exceptionalism. In R. G. de Almeida & L. R. Gleitman (Eds.), *On concepts, modules, and language: Cognitive science at its core* (pp.191-208). New York, NY: Oxford University Press.

Reichle, E. D., Rayner, K., & Pollatsek, A. (2003). The E-Z Reader model of eye-movement control in reading: Comparisons to other models. *Behavioral and Brain Sciences, 26*, 445-476.

Rousselet, G. A., Joubert, O. R., & Fabre-Thorpe, M. (2005). How long to get to the "gist" of real-world natural scenes? *Visual Cognition, 12*, 852-877.
