# Supplementary material for: Understanding Events by Eye and Ear: Agent and Verb Drive Non-anticipatory Eye Movements in Dynamic Scenes
Source: Front Psychol. 2019 Oct 10;10:2162. doi: 10.3389/fpsyg.2019.02162 (PMC6795699; doi:10.3389/fpsyg.2019.02162)
Supplement: Supplementary file 3 [file Data_Sheet_3.pdf]

Supplementary Material

Understanding Events by Eye and Ear: Agent and Verb Drive Non-Anticipatory Eye Movements in Dynamic Scenes

Roberto G. de Almeida[1*], Julia Di Nardo[1], Caitlyn Antal[1,2], Michael W. von Grünau[1]*

[1]Department of Psychology, Concordia University, Montreal, QC, Canada

[2]Department of Linguistics, Yale University, New Haven, CT, USA

*** Correspondence:**
Roberto G. de Almeida
roberto.dealmeida@concordia.ca

1 Scene Norms

We conducted a scene norming study with two goals in mind: (1) we wanted to gather information about the predictability of the scenes—in particular with regards to the event to take place involving agent and target object—and (2) we wanted to quantify the saliency of the target object as a function of (a) each movement condition and (b) its "informativeness" (Henderson & Hollingworth, 1999) as part of a naturalistic scene. Previous studies on scene processing have found that more informative objects within a scene—defined as objects that are not usually taken to "belong" to that scene (such as in Loftus & Mackworth's 1978 "octopus in a farm"/"tractor under water" case) draw more eye fixations during initial processing of the scene. Although Henderson, Weeks, and Hollingworth (1999) found no difference on first saccades to consistent and inconsistent objects in scenes, we wanted to determine the degree of salience of the target object to determine if there were differences across motion conditions.

In order to verify the predictability of the event as a function of different motion conditions, and to quantify the "informativeness" of target objects, we presented 34 Concordia University students with still frames of the movie triplets. The frames were selected from points in the movies where it appeared unambiguously that agents were about to perform a particular movement—e.g., to move away from a particular target object, or to move toward it, in the two critical cases—but without agents actually engaging objects or moving away from the scene (Figure 1, in the main text, represents one such triplet). These frames were distributed into three lists such that an equal number of away, toward and neutral conditions appeared in each list, but no slides representing the same scene appeared in each list. These slides were presented to each participant using Microsoft PowerPoint running automatically on a 17" Apple monitor. Each trial started with a fixation cross, followed by the presentation of the movie frame for 2 s. At the first 2 s of presentation of a still scene, Mackworth and Morandi (1967) found that informative regions receive most fixations. In Loftus and Mackworth's (1978) study, participants viewed a scene for 4 s. We predicted that 2 s would be sufficient for the purpose of determining the nature of the scene/event together with some

of its constituent objects. This is also in keeping with the findings that the "gist" of a scene can be extracted in a very short amount of time even in the absence of eye movements, thus indicating that objects and possibly event concepts might be processed at the earliest moments of the viewing of the scene (e.g., Potter, 2018; Thorpe, Fize, & Marlot, 1996; VanRullen & Thorpe, 2001). After each slide was presented, participants had to perform two tasks. First, they were instructed (via a 3 s slide) to "list the objects in the scene" by writing down their answers in a booklet. They had 15 s to list up to six objects (as marked in the booklet). Second, after listing the objects, they were presented with a tone and another slide instructing them to write down a sentence about the scene. In version *A* of the response booklet, the message was "What is about to happen in the scene?" and in version *B* it was "What is happening in the scene?" Again, participants had 15 s to record their responses, with the beginning and end times indicated by the same two tones.

For the word listing part of the task, we computed the relative frequency of the target object as a function of movement condition. This was computed by calculating the number of times the object was listed over the total number of objects listed across participants (thus, 1.0/6 = .167 would be the maximum relative frequency, since 6 was the maximum number of objects to be listed). A repeated-measures ANOVA was computed with motion type as the independent variable. The analysis indicated that there was a main effect of motion type, $F(2, 32) = 6.32$, $p < .01$. A modified Bonferroni/Dunn test, with adjusted alpha levels of .03 per pairwise comparison, was conducted to determine which conditions differed from each other. These comparisons revealed that the away ($M = .136$, $SD = .064$) and toward ($M = .156$, $SD = .050$) conditions differed significantly ($p = .02$), as did the neutral ($M = .127$, $SD = .065$) and toward conditions ($p < .01$). The away and neutral conditions were not significantly different $p = .31$). Notice that although the differences were significant, the magnitude of the relative frequencies shows that the target object received high attention across all conditions during the initial viewing of the still scenes.

For the sentence writing part of the task, we conducted two separate analyses. One for the *A* version of the booklet (*what is about to happen*) and another for the *B* version (*what is happening*). For both versions all sentences written by participants were coded as event structures, with predicate and arguments listed (e.g., [*break* [*the cook, eggs*]]). The relative frequency of predicates and arguments were computed for both *A* and *B* versions of the booklet. None of these analyses suggested that the events were predictable, as the range of target event matching the movies/scenes we created was 0-13.6% in the *A* version and 0-88.2% in the *B* version (with [*roll* [*child*, *cube*]] being the most predictable event). But there was no effect of motion condition in any of these versions of the booklet, $F_A\ (2, 32) = 1.15$, $p = .33$; $F_B\ (2, 28) < 1$, $p = .91$.

The results from these scene norming tasks indicate that target object saliency or "informativeness" and scene/event gist are balanced across conditions. It should be noted that, although the relative frequency of the target object in the toward condition was higher than in the other two conditions, the still scenes were not taken from the frames used to synchronize the verb onsets with the motion onsets, but from a later frame. Also, the magnitudes of the frequencies were all high for the three conditions. In addition, the event that *is about to happen* and that *is happening*, according to participants in the *A* and *B* conditions, respectively, are not predictable from the brief 2 s inspections of the scenes. It seems that any effects of context on linguistic processing and any eye-movement directed by verb properties should be taken as effects of the unfolding linguistic and visual context in the dynamic scenes.

Table S1

Target Object Listing Frequency

Target Object	Away	Neutral	Toward	Overall
Ball	.222	.207	.227	.213
Butter	.038	.097	.074	.071
Car Crash	.211	.189	.204	.201
Car Start	.204	.196	.194	.198
Chair	.138	.150	.170	.152
Cube[a]	.155	.217	.152	.179
Egg	.021	.037	.106	.054
Ice	.111	.033	.070	.068
Kite	.073	.038	.088	.067
Milk[b]	.128	.119	.143	.128
Oven[c]	.070	.042	.125	.084
Paper	.091	.080	.152	.106
Picture	.190	.157	.188	.178
Plate	.127	.158	.176	.153
Shirt	.123	.097	.158	.119
Shoes	.176	.140	.192	.169
Vase	.227	.200	.231	.221
Mean	.136	.127	.156	.139

[a]These numbers were computed by counting two separately listed objects: “box/square toy” (F[A] = .017, F[N] = .050, F[T] = .000, and F[Overall] = .026), which we took as referring to the cube, and “toy(s)” (F[A] = .138, F[N] = .167, F[T] = .152, and F[Overall] = .152), which was included because a cube belongs to the “toy” category.

[b]These numbers were computed by totalling the frequencies listed for both “milk” (F[A] = .021, F[N] = .000, F[T] = .024, and F[Total] = .014) and “cup” (F[A] = .106, F[N] = .119, F[T] = .119, and F[Overall] = .115), which referred to the same object.

[c]These numbers were computed by totalling the frequencies listed for both “oven” (F[A] = .000, F[N] = .021, F[T] = .063, and F[Overall] = .032) and “stove” (F[A] = .070, F[N] = .021, F[T] = .063, and F[Overall] = .052), which referred to the same object and were listed separately by one subject, precluding the lumping together of the two synonyms.

Table S2

Human Agent Listing Frequency

Scene	Away	Neutral	Toward	Overall
Ball	.222	.207	.182	.200
Butter	.173	.161	.167	.167
Car Crash	.123	.170	.184	.157
Car Start	.204	.176	.161	.179
Chair	.155	.167	.189	.170
Cube	.190	.250	.152	.205
Egg	.208	.185	.191	.195
Ice	.222	.183	.233	.209
Kite	.182	.212	.158	.183
Milk	.213	.186	.214	.203
Oven	.233	.188	.188	.200
Paper	.182	.200	.196	.192
Picture	.155	.176	.229	.185
Plate	.145	.123	.196	.153
Shirt	.158	.161	.158	.153
Shoes	.157	.211	.173	.181
Vase	.227	.250	.192	.221
Mean	0.185	0.189	0.186	0.185

Table S3

Target Event Propositional Structure Listing Frequency – Version "A" ("What will happen next?")

Scene	Away	Neutral	Toward	Overall
Ball	.000	.000	.000	.000
Butter	.000	.000	.000	.000
Car Crash	.000	.000	.000	.000
Car Start	.000	.000	.000	.000
Chair	.000	.000	.000	.000
Cube	.000	.000	.000	.000
Egg	.000	.000	.000	.000
Ice	.000	.000	.500	.150
Kite	.000	.091	.167	.083
Milk	.000	.000	.000	.000
Oven	.000	.000	.000	.000
Paper	.000	.000	.000	.000
Picture	.143	.000	.222	.107
Plate	.333	.000	.000	.136
Shirt	.000	.000	.000	.000
Shoes	.000	.000	.000	.000
Vase	.000	.000	.000	.000
Mean	0.028	0.005	0.052	0.028

Table S4

Target Event Propositional Structure Listing Frequency – Version "B" ("What is happening now?")

Scene	Away	Neutral	Toward	Overall
Ball	.000	.667	.000	0.222
Butter	.429	.167	.500	.333
Car Crash	.091	.100	.000	.077
Car Start	.000	.143	.100	.087
Chair	.400	.167	.125	.211
Cube	1.000	1.000	.600	.882
Egg	.333	.286	.500	.368
Ice	.750	.667	.000	.786
Kite	.333	.000	.125	.167
Milk	.286	.000	.200	.150
Oven	.400	.167	.200	.238
Picture	.000	.000	.000	.000
Shirt	.500	.667	.571	.600
Shoes	.429	.667	.500	.526
Vase	.800	.667	1.000	.824
Mean	0.383	0.357	0.361	0.365

References

Henderson J. M., & Hollingworth A. (1999). High-level scene perception. *Annual Review of Psychology*, *50*, 243-271.

Henderson J. M., Weeks P. A., & Hollingworth A. (1999). The effects of semantic consistency on eye movements during scene viewing. *Journal of Experimental Psychology: Human Perception and Performance*, *25*, 210-228

Loftus, G. R. & Mackworth, N. H. (1978). Cognitive determinants of fixation location during picture viewing. *Journal of Experimental Psychology: Human Perception and Performance*, *4*, 565-572.

Mackworth, N.H., & Morandi, A.J. (1967). The gaze selects informative details within pictures. *Perception & Psychophysics*, *2, 547–552.*

Potter, M. C. (2018). The immediacy of conceptual processing. In R. G. de Almeida & L. R. Gleitman (Eds.), *On concepts, modules, and language: Cognitive science at its core* (pp. 239-248). New York, NY: Oxford University Press.

Thorpe, S. J., Fize, D. & Marlot, C. (1996) Speed of processing in the human visual system. *Nature*, 381, 520-522

VanRullen, R. & Thorpe, S. J. (2001). Is it a bird? Is it a plane? Ultra-rapid visual categorization of natural and artifactual objects. *Perception, 30*, 655-668.
