# Supplementary material for: Understanding Events by Eye and Ear: Agent and Verb Drive Non-anticipatory Eye Movements in Dynamic Scenes
Source: Front Psychol. 2019 Oct 10;10:2162. doi: 10.3389/fpsyg.2019.02162 (PMC6795699; doi:10.3389/fpsyg.2019.02162)
Supplement: Supplementary file 4 [file Data_Sheet_4.pdf]

Supplementary Material

Understanding Events by Eye and Ear: Agent and Verb Drive Non-Anticipatory Eye Movements in Dynamic Scenes

Roberto G. de Almeida[1*], Julia Di Nardo[1], Caitlyn Antal[1,2], Michael W. von Grünau[1]*

[1]Department of Psychology, Concordia University, Montreal, QC, Canada

[2]Department of Linguistics, Yale University, New Haven, CT, USA

*** Correspondence:**
Roberto G. de Almeida
roberto.dealmeida@concordia.ca

1 Materials used in the eye-tracking experiment

Below are the seventeen scene triplets (Away, Neutral and Toward) and the corresponding sentence pairs used in the experiment. The verb before the forwardslash (/) is the more restrictive causative verb, while the second verb is the perception verb used in each sentence pair. Notice that some of the frames may not clearly show the direction of the agent, either because the motion was slow within the same path or because the picture is not clear regarding motion (torso or limbs) toward or away the object. In motion pictures the path is clearer. All videos are available upon request (http://psycholinguistics.weebly.com/materials/).

[Written informed consent was obtained from the depicted individuals shown in the pictures for their publication**]**

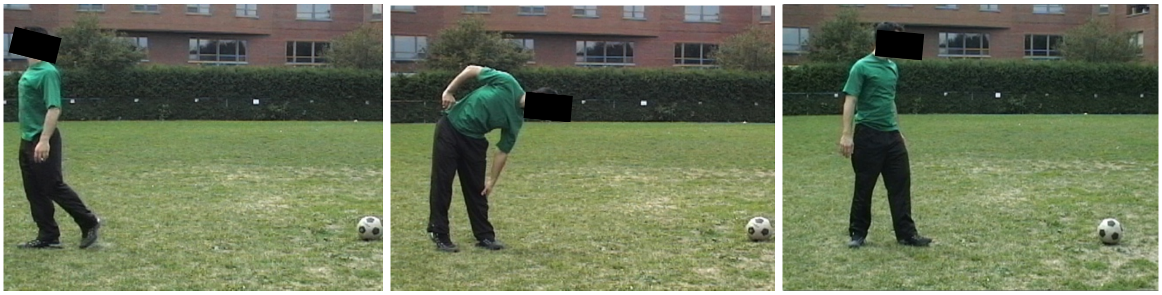

1. After his warm up, the athlete will drop/inspect the ball that he uses for drills.

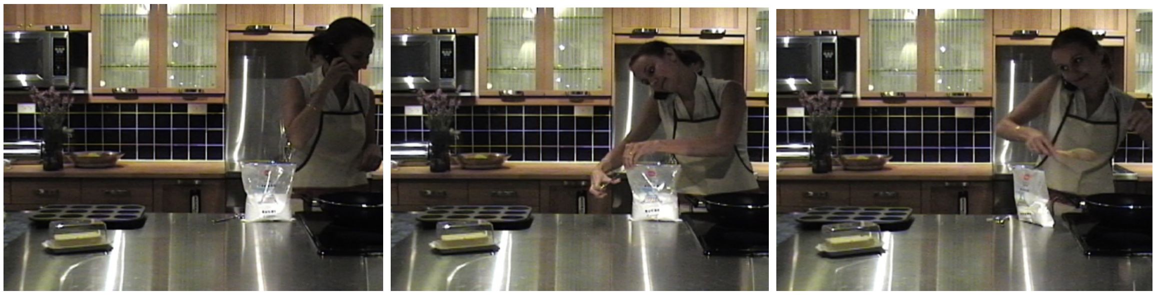

2. In order to bake some muffins, the woman will melt/check the butter that is required for the dough.

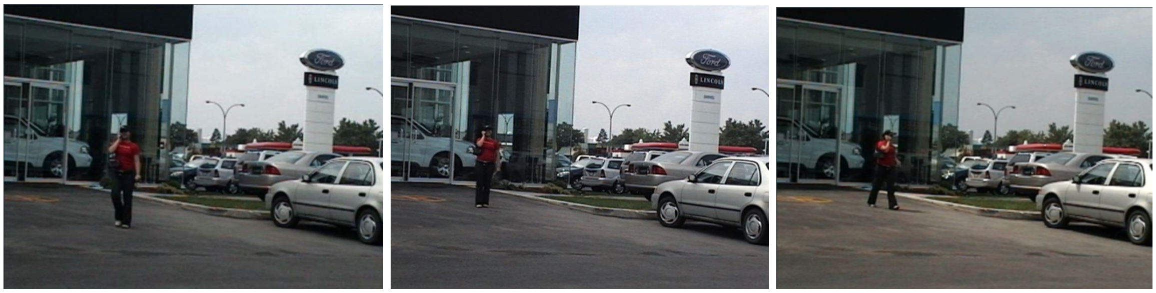

3. On her way to the station, the driver will crash/check the car that she just bought.

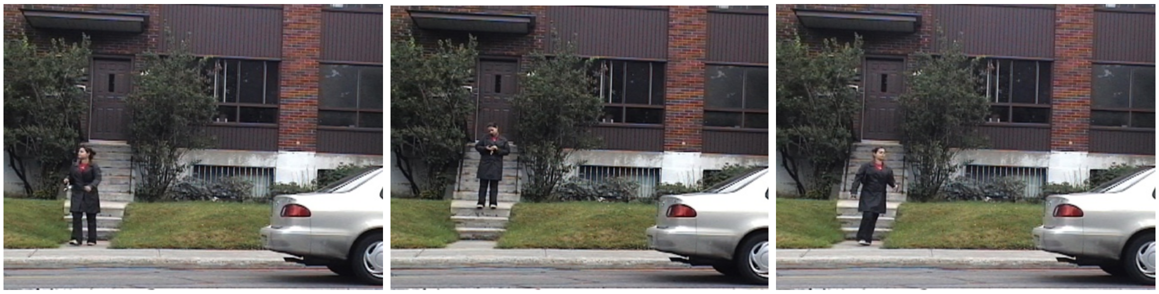

4. Before going to work, the driver will start/check the car that is in front of her house.

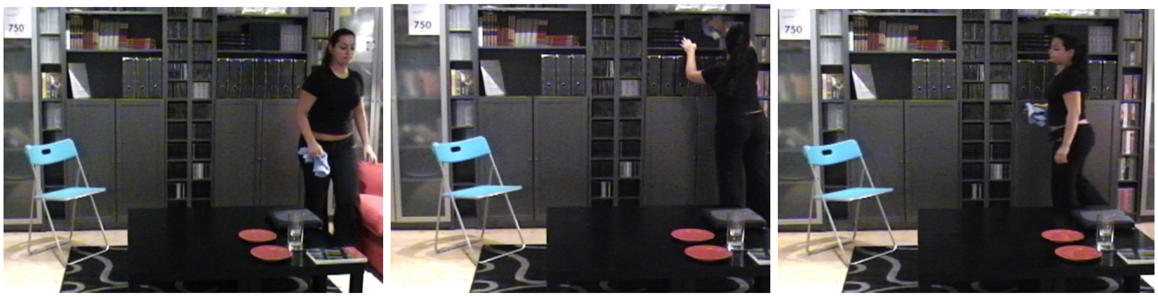

5. While dusting the furniture, the maid will fold/see the chair that is in the living room.

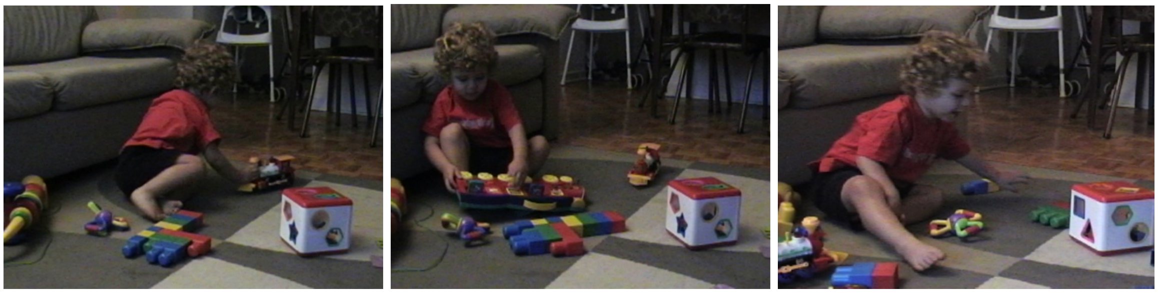

6. While playing with his toys, the infant will roll/notice the cube that is on the floor.

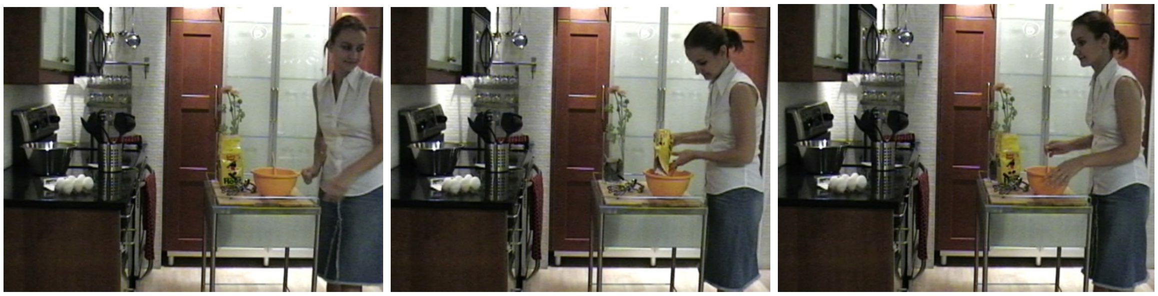

7. Before making the dessert, the cook will crack/examine the egg that is in the bowl.

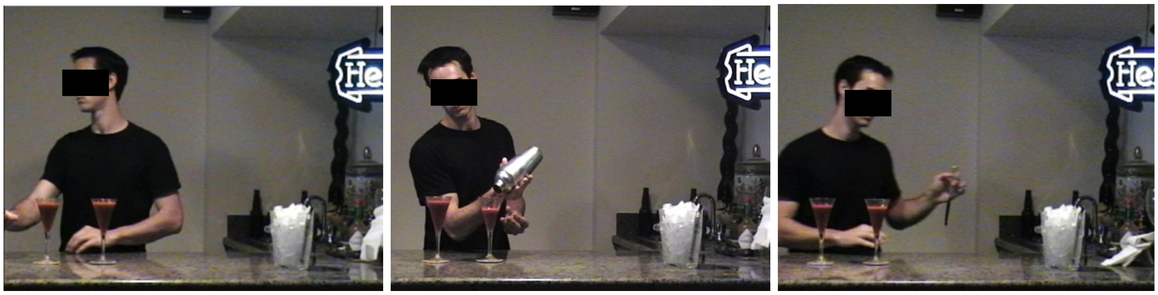

8. While preparing the drink, the bartender will crush/notice the ice that he has to put in the glass.

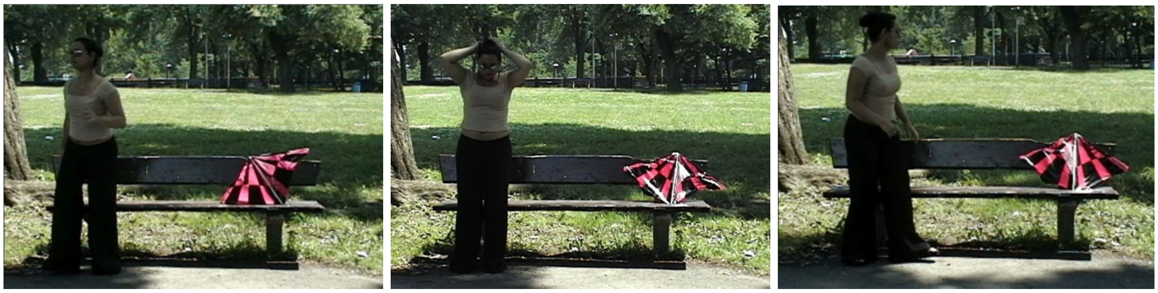

9. While standing in the park, the girl will fly/see the kite that is on the bench.

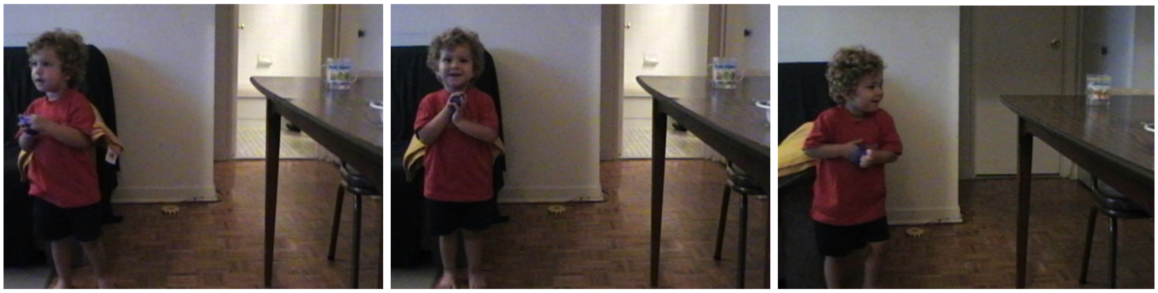

10. While playing with the lid, the child will spill/spot the milk that is on the table.

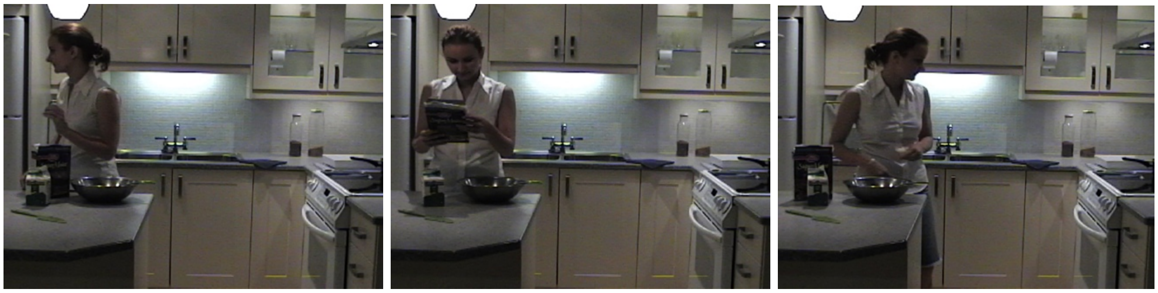

11. Before preparing the cake, the cook will heat/inspect the oven that is in the kitchen.

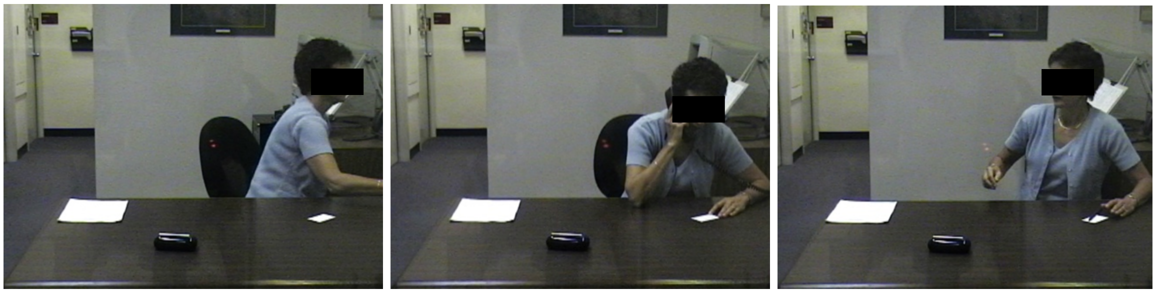

12. After talking on the phone, the secretary will rip/examine the paper that is on the desk.

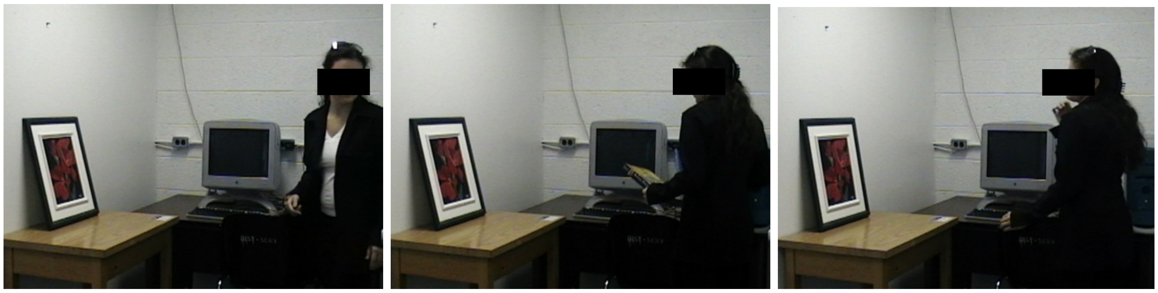

13. While unpacking her office, the student will hang/study the picture that she bought at the auction.

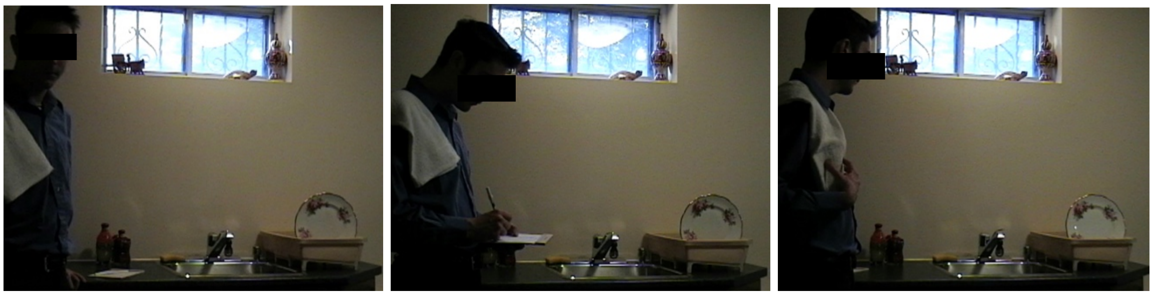

14. Before ending his shift, the busboy will dry/spot the plate that is on the counter.

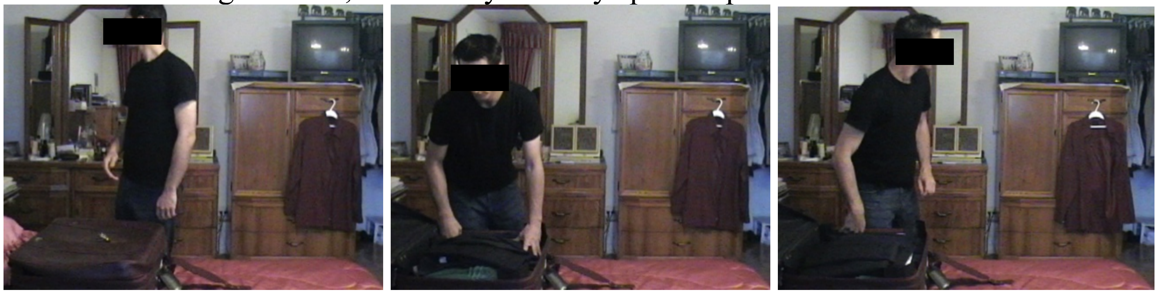

15. While packing his clothes, the man will wrinkle/see the shirt that he will use at the meeting.

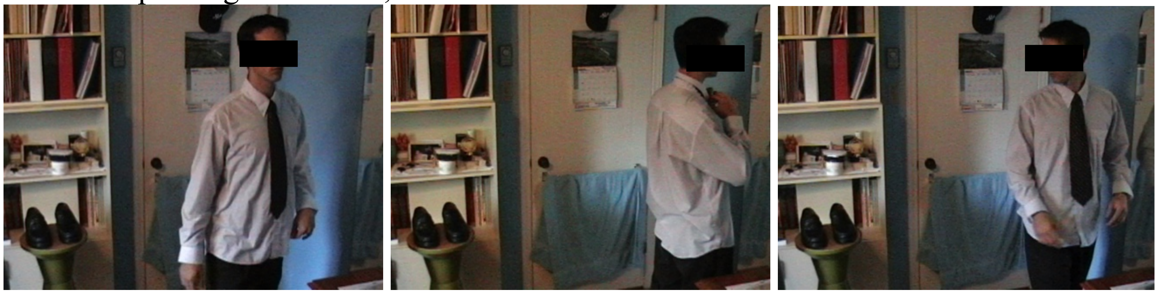

16. After getting ready for work, the businessman will shine/examine the shoes that he got from his wife.

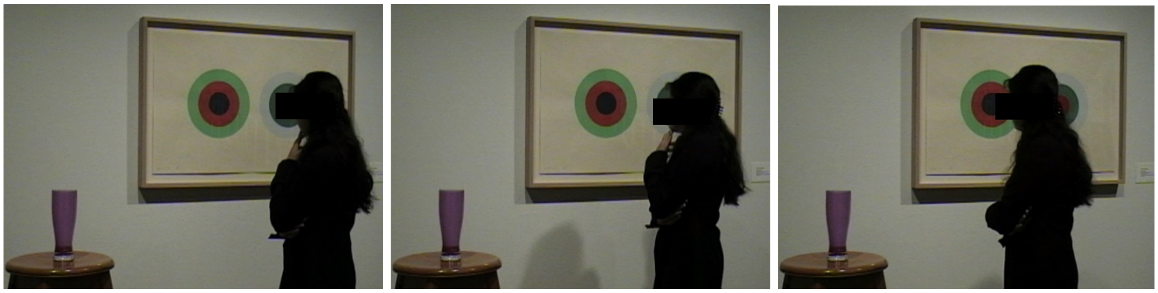

17. During her visit to the gallery, the girl will break/spot the vase that is on display.
